# Comparative Effectiveness of the Bivalent (Original/Omicron BA.4/BA.5) mRNA COVID-19 Vaccines mRNA-1273.222 and BNT162b2 Bivalent in Adults with Underlying Medical Conditions in the United States

**DOI:** 10.3390/vaccines12101107

**Published:** 2024-09-27

**Authors:** Hagit Kopel, Van Hung Nguyen, Alina Bogdanov, Isabelle Winer, Catherine Boileau, Thierry Ducruet, Ni Zeng, Jessamine P. Winer-Jones, Daina B. Esposito, Mary Bausch-Jurken, Ekkehard Beck, Machaon Bonafede, James A. Mansi

**Affiliations:** 1 Moderna, Inc., Cambridge, MA 02139, USA; hagit.kopel@modernatx.com (H.K.); daina.esposito@modernatx.com (D.B.E.); mary.bausch-jurken@modernatx.com (M.B.-J.); ekkehard.beck@modernatx.com (E.B.); 2 VHN Consulting Inc., Montreal, QC H2V 3L8, Canada; vhnguyen@vhnconsulting.com (V.H.N.); cboileau@vhnconsulting.com (C.B.); thierryducruet@vhnconsulting.com (T.D.); 3 Veradigm, Chicago, IL 60654, USA; alina.bogdanov@veradigm.com (A.B.); ni.zeng@veradigm.com (N.Z.); jessamine.winer-jones@veradigm.com (J.P.W.-J.); mac.bonafede@veradigm.com (M.B.)

**Keywords:** COVID-19, vaccine, mRNA-1273, BNT162b2, high risk, comorbidities, rVE

## Abstract

Background/Objectives: This retrospective cohort study evaluated the relative vaccine effectiveness (rVE) of two bivalent (original/Omicron BA.4/BA.5) vaccines mRNA-1273.222 versus the BNT162b2 Bivalent in preventing COVID-19-related outcomes in adults with underlying medical conditions associated with increased risk for severe COVID-19. Methods: In a linked electronic health record/claims dataset, US adults (≥18 years) with ≥1 underlying medical condition of interest who received either the bivalent vaccine between 31 August 2022 and 28 February 2023 were identified. The inverse probability of treatment weighting was used to adjust for cohort differences. Cohorts were followed up for COVID-19-related hospitalizations and outpatient encounters until 31 May 2023. Hazard ratios and rVEs were estimated using Cox regression. Subgroup analyses were performed on individuals with pre-specified comorbid conditions. Results: 757,572 mRNA-1273.222 and 1,204,975 BNT162b2 Bivalent recipients were identified. The adjusted rVE over a median follow-up of 198 days was 10.9% (6.2%–15.2%) against COVID-19-related hospitalization and 3.2% (1.7%–4.7%) against COVID-19-related outpatient encounters. rVE estimates for COVID-19 hospitalizations among subgroups with comorbid conditions were as follows: diabetes 15.1% (8.7%–21.0%), cerebro- and cardiovascular disease 14.7% (9.0%–20.1%), chronic lung disease 11.9% (5.1%–18.2%), immunocompromised 15.0% (7.2%–22.2%), chronic kidney disease 8.4% (0.5%–15.7%). Conclusions: Overall, among adults with underlying medical conditions, mRNA-1273.222 was more effective than BNT162b2 Bivalent, especially in preventing COVID-19-related hospitalizations.

## 1. Introduction

Since its emergence in late 2019, the SARS-CoV-2 virus has been continuously evolving and diverging from the strain used to develop the original COVID-19 vaccines [1]. The emergence of the Omicron variant and subvariants, with their ~50 mutations that promote transmission and immune evasion, resulted in reduced vaccine protection and increased disease burden, including higher infection and hospitalization rates [2,3].

With the aim of targeting the predominant circulating variants during the Omicron period, bivalent mRNA vaccines, which included mRNA from the original (wild-type) SARS-CoV-2 strain, together with an Omicron subvariant (BA.4/BA.5), were developed [4,5]. The vaccines were authorized for use in the US by the US Food and Drug Administration (FDA) in August 2022 and recommended by the US Centers for Disease Control and Prevention (CDC) for all adults in September 2022 [6].

Although all individuals are susceptible to severe outcomes following SARS-CoV-2 infection, some groups, such as older adults and select racial and ethnic minorities, were identified early in the pandemic as being at a higher risk of severe outcomes [7,8]. In addition, individuals with specific underlying medical conditions were shown to be at an increased risk for severe outcomes. The list of underlying medical conditions associated with increased risk includes immunocompromised conditions, cerebro- and cardiovascular diseases, diabetes, and obesity, some of which are highly prevalent in the US adult population, including young adults. An analysis conducted on the National Health Interview Survey (NHIS) has shown that more than 50% of the US adult population (>18) has been diagnosed with at least one chronic condition [9]. Notably, the majority of US adult individuals (including young adults) hospitalized with COVID-19 had at least one underlying medical condition before and during the Omicron period [10,11]. Thus, optimizing protection against COVID-19-related outcomes, especially among these high-risk populations, has direct public health implications which can reduce the burden in terms of infections, chronic complications, and severe outcomes.

While the two bivalent (original/Omicron BA.4/BA.5) mRNA vaccines that were available in the US (mRNA-1273.222, developed by Moderna and BNT162b2 Bivalent, developed by Pfizer BioNTech) during the 2022–2023 season used a similar vaccine technology, previous analysis has shown that mRNA-1273.222 was more effective than BNT162b2 Bivalent in adults aged ≥ 18 years old [12]. In this analysis, we used the same methodology to evaluate the relative vaccine effectiveness (rVE) of mRNA-1273.222 versus the BNT162b2 Bivalent vaccine in the prevention of COVID-19-related hospitalization and outpatient encounters in adult patients (≥18 years of age) living in the US with ≥1 underlying medical condition associated with an increased risk of severe outcomes from COVID-19.

## 2. Methods

### 2.1. Data Source

This study leveraged electronic health record (EHR) data from the Veradigm Network EHR linked to healthcare claims sourced from Komodo Health spanning 1 March 2020 through 31 May 2023. This integrated dataset has been previously characterized and used previously in COVID-19 epidemiology and VE research [10,12,13,14]. The dataset used in this study contains only de-identified data as per the de-identification standard defined in Section §164.514(a) of the Health Insurance Portability and Accountability Act of 1996 (HIPAA) Privacy Rule. As a noninterventional, retrospective database study using data from a certified HIPAA-compliant de-identified research database, approval by an institutional review board was not required.

The Komodo data contain claims sourced directly from payers as well as other sources, such as revenue cycle management platforms and claims clearinghouses. For the purpose of this analysis, the closed claims analysis leveraged all claims captured during a period of continuous health plan enrollment, regardless of the claim source. By contrast, the open claims sensitivity analysis leveraged all claims during the study period regardless of health plan enrollment. When multiple claims appeared for the same encounter, the claim from the payer superseded the claims from any other source.

### 2.2. Study Design and Study Population

This retrospective, observational cohort study was designed, implemented, and reported in accordance with Good Pharmacoepidemiological Practice, applicable local regulations, and the ethical principles laid down in the Declaration of Helsinki.

**Figure 1:**
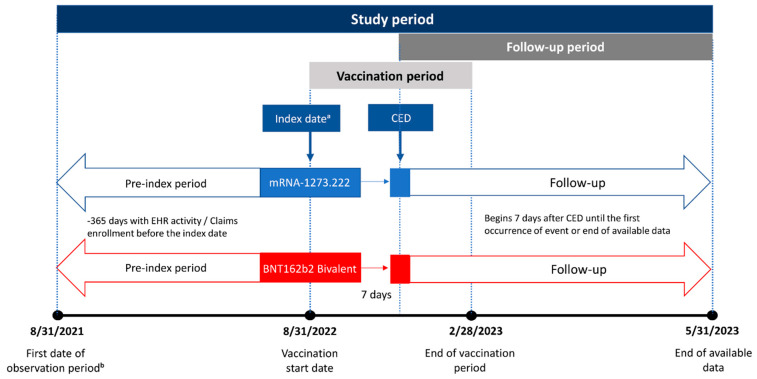
Study design. CED, cohort entry date. ^a^ The index date is the vaccination date. ^b^ Begins 365 days before vaccination with a bivalent vaccine.

Individuals ≥ 18 years of age with ≥1 underlying medical condition associated with an increased risk of severe outcomes from COVID-19 as defined by the CDC [15] who had received either the mRNA-1273.222 (50 mcg) or BNT162b2 Bivalent vaccine (30 mcg) between 31 August 2022 and 28 February 2023 were eligible for inclusion in the study. Underlying medical conditions were identified based on medical encounters in the 365 days before vaccination. Codes used to identify the underlying medical conditions are listed in Supplementary Table S1. Codes used to identify vaccination with bivalent mRNA vaccines are reported in Kopel et al. [12]. The index date was defined as the date of receipt of the bivalent vaccine, and the cohort entry date (CED) was defined as 7 days after the index date (Figure 1). The follow-up period for outcomes of interest for each individual participant began on the CED and continued until the first occurrence of an event of interest, disenrollment from their medical/pharmacy plan, the receipt of an additional COVID-19 vaccination, or the end of available data (31 May 2023), whichever occurred first. An end date of 31 May 2023 allowed for the possibility of up to three months of follow-up on individuals vaccinated on the last day of the vaccination window.

Individuals were included in the main analysis if they had a minimum of 365 days of continuous medical and pharmacy claims enrollment in a contributing health plan and ≥1 contact with a health service provider in the previous 365 days. Patients with evidence of COVID-19 infection or additional vaccination between the index date and CED, <1 day of follow-up available, or missing birth year/sex were excluded. The two cohorts were mutually exclusive, and the assignment was based on the first vaccine received during the vaccination period.

### 2.3. Objectives

The primary objective of this study was to assess the rVE of mRNA-1273.222 versus BNT162b2 Bivalent in preventing COVID-19-related hospitalization. The secondary objective was to assess the rVE of mRNA-1273.222 versus BNT162b2 Bivalent in preventing COVID-19-related outpatient encounters. Both primary and secondary objectives included individuals with any CDC-defined underlying medical condition associated with an increased risk of severe outcomes from COVID-19.

The exploratory objective of this study was to assess the primary and secondary objectives (rVE of COVID-19-related hospitalizations and outpatient encounters) among 5 pre-specified subgroups of patients with underlying medical conditions: cerebro- and cardiovascular, chronic kidney disease, chronic lung disease, diabetes, and immunocompromised. These five groups were selected based on their prevalence among the adult population and the risk for severe outcomes [10,15,16]. The specific underlying medical conditions included in these subgroups are defined in Table 1.

### 2.4. Outcome Measures

The primary outcome of interest was COVID-19-related hospitalizations, which were identified from hospitalization claims with documentation of COVID-19 diagnosis in any position. The secondary outcome of interest was COVID-19-related outpatient encounters, which were identified from outpatient EHR and medical claims with a COVID-19 diagnosis. Outpatient encounters included visits in various clinical settings, such as emergency department visits, urgent care visits, office visits, and telemedicine visits. The codes used to identify outcomes are reported in Kopel et al. [12].

### 2.5. Covariates

The study utilized patient characteristics as covariates to adjust for any baseline differences between vaccine cohorts (see the statistical analysis). The following demographic variables were captured at the index date: age, sex, race, ethnicity, insurance type, region, and month of index. COVID-19 vaccination and infection history were assessed using a lookback period that started on 1 March 2020 and included primary-series vaccination, time since the last COVID-19 monovalent vaccination in months, time since the last COVID-19 infection in months, and the data source of the recorded vaccination. Healthcare resource utilization variables were assessed during the 365-day baseline period and included the number of prior hospitalizations and the number of prior outpatient encounters.

The following underlying medical conditions were captured in the 365 days prior to, and inclusive of, the index date: asthma, cancer, cerebrovascular disease, chronic kidney disease, chronic liver disease, chronic lung disease, cystic fibrosis, dementia, diabetes mellitus, disability, heart conditions, human immunodeficiency virus (HIV), mental health conditions, obesity (body mass index > 30), physical inactivity, primary immunodeficiencies, respiratory tuberculosis, smoking, solid organ or stem cell transplant, and use of select immunosuppressive medications. Pregnancy was captured in the 301 days following the index date. The codes used to identify patients with underlying medical conditions are listed in Supplementary Table S1.

### 2.6. Statistical Analysis

Statistical analysis followed the methodology described in Kopel et al. [12]. Briefly, propensity scores predicting the receipt of mRNA-1273.222 or BNT162b2 Bivalent were calculated using a multivariable logistic model, adjusting for all covariates outlined above. Stabilized and truncated weights were used to re-weight the study sample using the inverse probability of treatment weighting (IPTW) methodology. A 1% asymmetrical trim was used to limit the effects of outlier weights on the study sample. Standardized mean differences (SMDs) were calculated to assess sample balance before and after IPTW. SMDs with absolute values > 0.1 indicated covariate imbalance. The method of calculating the SMDs for continuous and categorical variables is reported in the Supplementary Material.

Covariates were reported descriptively before and after weighting. Mean and standard deviation (SD) are reported for continuous variables, while number (N) and percent are reported for categorical variables. Follow-up time in days is captured and reported as the median and interquartile range (IQR).

Unadjusted hazard ratios (HRs) were reported for the unweighted sample, and adjusted HRs were reported for the weighted sample. Unadjusted HRs were estimated using Cox regression models, with exposure as the only predictor. Adjusted HRs were estimated using a multivariable Cox regression model that included the exposure and any baseline covariates with a SMD > 0.1 after weighting. The rVEs were calculated as 100 × (1 − HR) for both unadjusted and adjusted estimates with 95% confidence intervals (95% CIs).

All analyses were performed in the overall population of adults ≥ 18 years of age with ≥1 underlying medical condition to address the primary objective and secondary objective and then repeated separately for each of the prespecified five cohorts with specific underlying medical conditions of interest (cerebro- and cardiovascular disease, chronic kidney disease, chronic lung disease, diabetes, and immunocompromised). To control the inflation of the type 1 error rate due to multiple testing (family-wise error rate), we used a step-down testing procedure for secondary endpoints and the sensitivity analyses by adjusting the rejection criteria for each of the individually tested hypotheses. We conducted statistical testing at a significance level of *p* = 0.05 and stopped when a secondary endpoint did not reach statistical significance.

All statistical analyses were performed using SAS 9.4 or the R Statistical Software (v4.1.3) survival (v3.2-13) package.

### 2.7. Sensitivity Analyses

The main analysis was restricted to patients with more than 365 days of continuous health plan enrollment with medical and pharmacy benefits. As this approach may be biased towards patients with stable health insurance, we conducted a sensitivity analysis of the primary and secondary objectives using an open claims approach. The methodology used was the same as the main analysis, except there were no requirements for continuous health plan enrollment. Instead, we required that individuals have at least one medical or pharmacy claim in the 365 days preceding the index date.

A second sensitivity analysis examined the primary and secondary objectives in a shorter follow-up period ending on 28 February 2023. Apart from the shorter follow-up period, all analytical methods were consistent with the main analysis.

The magnitude and direction of the primary outcomes in the sensitivity analyses were compared to those of the main analysis to identify any potential bias from the claims source or follow-up duration.

## 3. Results

**Figure 2:**
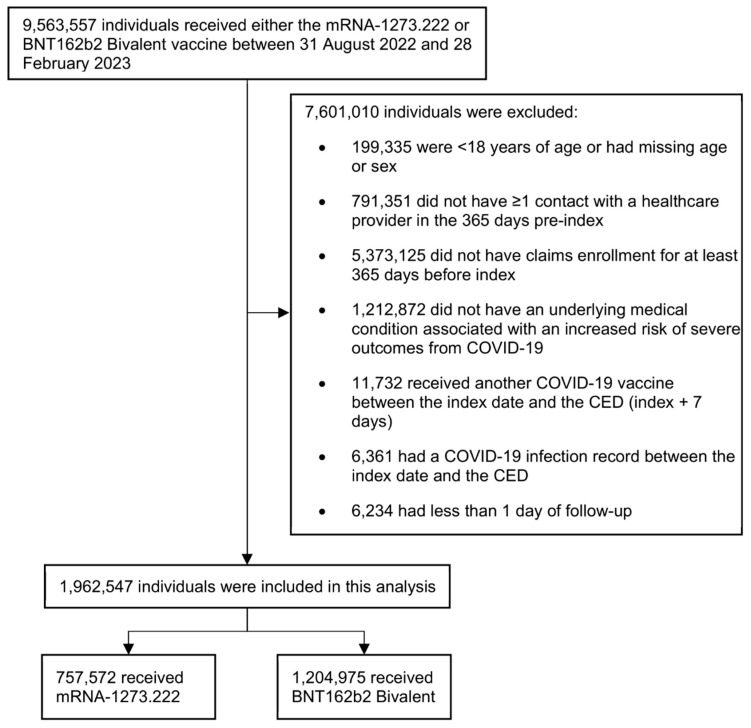
Selection of participants for inclusion in the study. CED, cohort entry date.

We identified 1,962,547 adults ≥ 18 years old with at least one underlying medical condition who received a bivalent mRNA vaccine between 31 August 2022 and 28 February 2023 and met all other study criteria (Figure 2). Of these, 757,572 (38.6%) received the mRNA-1273.222 vaccine, and 1,204,975 (61.4%) received the BNT162b2 Bivalent vaccine. Before weighting, the mean (SD) age was 62 (16) years in the mRNA-1273.222 cohort and 60 (16) years in the BNT162b2 Bivalent cohort, and 56.9% and 57.9% of the cohort were female, respectively (Table 2). The most common underlying medical conditions in both cohorts were obesity (mRNA-1273.222: 33.7%; BNT162b2 Bivalent: 34.1%), diabetes (mRNA-1273.222: 33.3%; BNT162b2 Bivalent: 32.2%), and mental health disorders (mRNA-1273.222: 26.7%; BNT162b2 Bivalent: 28.4%). The median (IQR) duration of follow-up was 197 days (147–225 days) for the mRNA-1273.222 cohort and 200 (148–228) for the BNT162b2 Bivalent cohort.

Before weighting, the subgroup analyses included 640,511 individuals with diabetes, 516,081 with cerebro- and cardiovascular disease, 474,573 with chronic lung disease, 427,652 who were immunocompromised, and 270,582 with chronic kidney disease (Table 3). This comprised 33.3% of the mRNA-1273.222 cohort and 32.2% of the BNT162b2 Bivalent cohort who were included in the diabetes subgroup analysis, 27.1% and 25.8% who were included in the cerebro- and cardiovascular disease subgroup analysis, 24.2% and 24.1% who were included in the lung disease subgroup analysis, 22.3% and 21.4% who were included in the immunocompromised subgroup analysis, and 14.1% and 13.6% who were included in the chronic kidney disease subgroup analysis, respectively. These subgroups were not mutually exclusive, as individuals may have had multiple underlying medical conditions. The median follow-up time in the subgroups ranged from 195 to 199 in the mRNA-1273.222 cohort and from 197 to 202 in the BNT162b2 Bivalent cohort.

Baseline characteristics of the overall analyses and the subgroup analyses before and after weighting are reported in Table 2 and Supplementary Tables S2–S6. After weighting, no variables had an SMD > 0.1 in either the overall study cohorts or in the underlying medical condition subgroups.

### 3.1. Main Analysis

In the overall study cohort, post weighting, 2360 (0.31%) individuals who received mRNA-1273.222 and 4198 (0.35%) who received BNT162b2 Bivalent were hospitalized for COVID-19 during the follow-up period. Conversely, 26,185 (3.5%) individuals who had received mRNA-1273.222 and 42,866 (3.6%) who had received BNT162b2 Bivalent had an outpatient encounter related to COVID-19.

**Figure 3:**
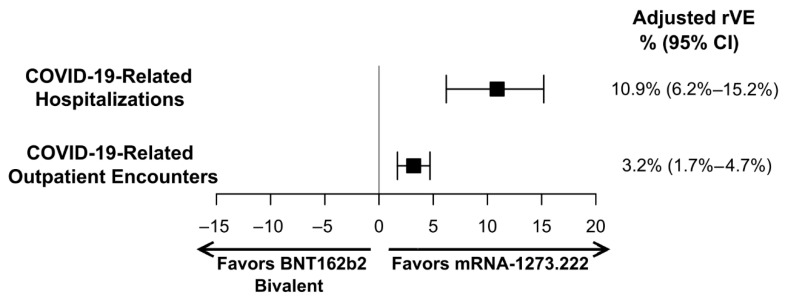
Adjusted relative vaccine effectiveness (rVE) estimates of mRNA-1273.222 vs. BNT162b2 Bivalent for adults with ≥1 underlying medical condition. CI, confidence interval.

For both the primary and secondary endpoints, mRNA-1273.222 was significantly more effective at preventing the outcomes of interest compared to BNT162b2 Bivalent. Specifically, the rVE (95%CI) against COVID-19-related hospitalization was 10.9% (6.2%–15.2%, *p* < 0.001), and the rVE against outpatient COVID-19 was 3.2% (1.7%–4.7%, *p* < 0.001) (Figure 3).

**Figure 4:**
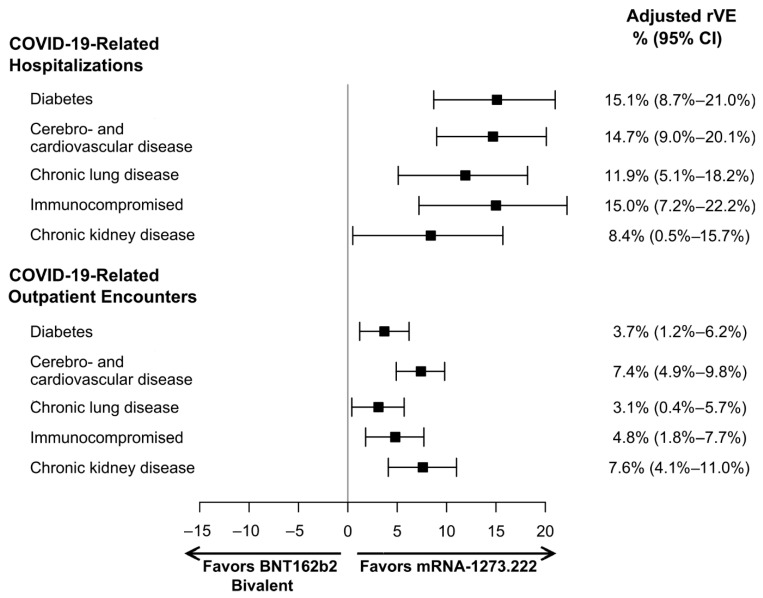
Adjusted relative vaccine effectiveness (rVE) estimates of mRNA-1273.222 versus BNT162b2 Bivalent among subgroups with specific underlying medical conditions. CI, confidence interval.

For all subgroups, mRNA-1273.222 was significantly more effective at preventing COVID-19-related hospitalizations and outpatient encounters compared to BNT162b2 Bivalent (Figure 4). The rVE (95% CI) of mRNA-1273.222 vs. BNT162b2 Bivalent against COVID-19-related hospitalization was 15.1% (8.7%–21.0%) in patients with diabetes, 14.7% (9.0%–20.1%) in those with cerebro- and cardiovascular disease, 11.9% (5.1%–18.2%) in those with chronic lung disease, 15.0% (7.2%–22.2%) in immunocompromised patients, and 8.4% (0.5%–15.7%) in CKD patients. Point estimates for rVE against outpatient COVID-19 were lower but statistically significant and ranged from 3.1% in immunocompromised patients to 7.6% in patients with CKD. Unadjusted and adjusted hazard ratios and 95% CIs are reported in Table 4. Unadjusted rVEs are reported in Supplementary Table S7.

### 3.2. Sensitivity Analyses

The results of the sensitivity analysis were consistent with the findings of the main analysis (Table 4 and Table 5). Specifically, in the open claims dataset, we identified 1,960,185 adults ≥ 18 years old with at least one underlying medical condition who received mRNA-1273.222 and 2,969,597 who received the BNT162b2 Bivalent vaccine during the vaccination period. In this population, the rVE (95% CI) of mRNA-1273.222 versus BNT162b2 Bivalent against COVID-19-related hospitalization was 12.0% (9.3%–14.7%), and the rVE against outpatient COVID-19 was 5.0% (4.0%–5.9%). Similarly, the use of a shorter follow-up period in the closed claims database resulted in rVE estimates of 13.1% (7.8%–18.2%) and 3.8% (2.1%–5.4%) against COVID-19-related hospitalization and COVID-19-related outpatient encounters, respectively. Baseline characteristics of individuals included in the sensitivity analyses before and after weighting are reported in Supplementary Tables S8 and S9.

## 4. Discussion

In this real-world retrospective analysis of more than 1.9 million individuals vaccinated with a bivalent COVID-19 vaccine, mRNA-1273.222 was significantly more effective than BNT162b2 Bivalent in preventing COVID-19-related hospitalizations in adults ≥ 18 years old with at least one underlying medical condition. Similarly, mRNA-1273.222 offered greater protection against outpatient encounters compared to BNT162B2 Bivalent but to a lesser extent. These results were consistent in the subgroup analyses of prespecified comorbid conditions, including diabetes, cerebro- and cardiovascular disease, immunocompromised conditions, chronic lung disease, and chronic kidney disease. Notably, the median follow-up duration was greater than 6 months, and the results were similar in the sensitivity analysis of the shorter follow-up duration. As SARS-CoV-2 continues to evolve, this study highlights the need to reassess vaccine strategies to provide optimal protection for everyone and especially for adults with underlying medical conditions who require comprehensive clinical management and are at a higher risk of severe outcomes.

These findings align with our previous analysis of the relative effectiveness (rVE) of bivalent vaccines in the overall adult population. We reported rVEs (95% CI) of 9.8% (2.6–16.4%) against COVID-19-related hospitalizations and 5.1% (3.2%–6.9%) against COVID-19-related outpatient encounters for mRNA-1273.222 compared to BNT162b2 Bivalent [14]. Among adults aged 65 and older, the estimated rVEs were slightly higher, showing 13.5% (5.5%–20.8%) against COVID-19-related hospitalizations and 10.7% (8.2%–13.1%) against COVID-19-related outpatient encounters.

Earlier studies assessing the primary-series and initial monovalent boosters found similar results, showing greater effectiveness of the mRNA-1273 vaccine over the BNT162b2 vaccine, particularly in groups at higher risk due to age and comorbidities [14,17,18,19]. These findings have remained consistent across different variant periods for high-risk groups and extend to subpopulations with specific medical conditions. For instance, vaccine effectiveness (VE) against infection and death was higher for the primary series of mRNA-1273 compared to BNT162b2 in a study focusing on individuals with diabetes. Similarly, the effectiveness against medically attended SARS-CoV-2 infection or related hospitalization after a third dose was higher in patients with diabetes, hypertension, heart disease, cancer, or COPD [20,21].

The difference in protection afforded by the vaccines may be a result of a stronger immune response elicited by mRNA-1273 compared to BNT162b2, especially in individuals who are immunocompromised or have comorbid conditions. In a pairwise meta-analysis across various immunocompromising conditions, the mRNA-1273 vaccination was more likely to result in seroconversion and higher antibody titers than the BNT162b2 vaccination [22]. Additionally, studies have shown a stronger humoral response associated with the mRNA-1273 vaccination in type 1 diabetics, dialysis patients, patients with chronic medical conditions, and elderly individuals with multiple comorbid conditions compared to the BNT162b2 vaccination [23,24,25,26].

Research has established that individuals with certain underlying medical conditions represent approximately 22% of the adult global population [27]. These individuals are at an increased risk of severe outcomes from COVID-19 [28,29,30], and this risk increases further with the number of conditions [31]. An analysis of a large US hospital all-payer database found that the risk ratio (95% CI) of death among individuals hospitalized with COVID-19 increased from 1.5 (1.4–1.7) for individuals with 1 underlying medical condition to 3.8 (3.5–4.2) for individuals with more than 10 underlying medical conditions relative to individuals without any underlying medical conditions [31]. As expected, severe COVID-19 outcomes increase the economic burden. Specifically, hospitalization costs for COVID-19 patients with comorbid conditions have been significantly higher when compared to individuals without known underlying conditions. Patients with chronic kidney disease had a 64% higher cost, liver disease patients had a 37% higher cost, and those with cerebrovascular disease had a 30% higher cost compared to those without these conditions [32]. In addition, following the acute phase of COVID-19, patients at a higher risk of COVID-19 were also found to have increased overall healthcare spend and cost. A study using US commercial and Medicare Advantage claims of patients at high risk for COVID-19 estimated the mean overall per-patient medical cost during the year following acute COVID-19 at USD 27,077 which represents an increase of USD 5200 or 23.8% in comparison to the medical cost occurring in the year before the COVID-19 infection [33]. Thus, COVID-19 contributes to increasing the already high non-COVID healthcare costs associated with patients at high risk.

In summary, maximizing protection against preventive diseases, especially among patients with underlying medical conditions who require comprehensive clinical management, can reduce the burden of COVID-19 on individuals and the healthcare system.

### Strengths and Limitations

The primary strength of this analysis is the depth and breadth of the linked EHR and claims dataset used in this analysis. By integrating sources of patient information across the continuum of care, we increase the likelihood of capturing exposures (vaccination events), covariates (patient characteristics and underlying medical conditions), and outcomes (COVID-19-related hospitalizations and outpatient encounters). Additionally, as information on exposure, outcome, and covariates was collected from patient records in a consistent manner across all cohorts, there is a reduced likelihood of differential misclassification. However, there were baseline differences in the two cohorts predominantly in the type of primary-series vaccination and the time since the last COVID-19 vaccination. This residual confounding between cohorts was addressed using propensity score weighting and multivariable regression.

This study is subject to several limitations which are inherent to many non-interventional studies utilizing real-world data. Specifically, this retrospective observational study, which relies on routinely collected data on insured patients, might include data entry errors and cannot be generalized to the uninsured population. Additionally, the current primary analysis was restricted to closed claims, which provide a comprehensive overview of a patient’s healthcare interactions but may not capture all cases and is restricted to patients with stable healthcare insurance. However, the sensitivity analysis performed using an open claims approach was consistent with the main analysis, which supports the broad generalizability of our findings. Furthermore, we required that study subjects had at least one healthcare encounter during the previous 365 days, which would have excluded healthy patients; however, as this study was restricted to individuals with underlying medical conditions, the effects of this bias were minimized.

Finally, there may have been differences between the two vaccine groups that were not fully accounted for by the pre-defined covariates and residual confounding may be present between the cohorts. For example, differences in the timing and availability of the two vaccines may have impacted which patients were more likely to receive one vaccine over the other; however, the prevalence of individual high-risk conditions was similar between the two cohorts even before weighting. In our previous analysis that included the overall adult population, we found no difference in the proportion of patients vaccinated with each vaccine over time [12].

## 5. Conclusions

In this real-world analysis, mRNA-1273.222 was significantly more effective than BNT162b2 Bivalent at preventing COVID-19-related hospitalizations and outpatient encounters among adults ≥ 18 years with at least one underlying medical condition associated with higher risk for COVID-19 severe outcomes.

## Figures and Tables

**Table 1:** Underlying medical conditions included in the subgroup analyses.

Subgroup	Conditions ^a^
Diabetes	Diabetes type 1 or type 2
Cerebro- and cardiovascular	Cerebrovascular disease, heart disease
Chronic lung disease	Asthma, other chronic lung diseases (e.g., pulmonary heart diseases, chronic obstructive pulmonary disease, and bronchiectasis)
Immunocompromised	Cancer, human immunodeficiency virus, primary immunodeficiencies/other immunocompromising conditions (e.g., hereditary hypogammaglobulinemia, selective deficiency of immunoglobulins, severe combined immunodeficiencies, and common variable immunodeficiencies), solid organ or blood stem cell transplantation, stem cell transplantation, use of immunosuppressive medications
Chronic kidney disease	Chronic kidney disease

^a^ The full list of diagnoses used to define these condition lists are included in Supplementary Table S1.

**Table 2:** Key baseline characteristics of adults with ≥1 underlying medical condition included in the mRNA-1273.222 and BNT162b2 Bivalent vaccine cohorts (pre- and post weighting).

		Pre-Weighting			Post Weighting		
		mRNA-1273.222	BNT162b2 Bivalent	SMD	mRNA-1273.222	BNT162b2 Bivalent	SMD
Number of patients		757,572	1,204,975		758,803	1,202,211	
Age at index in years, mean (SD)		62 (16)	60 (16)	0.096	61 (16)	61 (16)	0.003
Sex, N (%)	Female	431,238 (56.9)	697,587 (57.9)	0.020	436,546 (57.5)	691,694 (57.5)	<0.001
	Male	326,334 (43.1)	507,388 (42.1)		322,257 (42.5)	510,518 (42.5)	
Race, N (%)	Black	47,522 (6.3)	82,125 (6.8)	0.031	49,881 (6.6)	79,383 (6.6)	0.001
	Other	39,457 (5.2)	60,368 (5.0)		38,699 (5.1)	61,213 (5.1)	
	White	379,026 (50.0)	589,334 (48.9)		374,392 (49.3)	592,955 (49.3)	
	Unknown	291,567 (38.5)	473,148 (39.3)		295,831 (39.0)	468,661 (39.0)	
Ethnicity, N (%)	Hispanic	37,723 (5.0)	63,334 (5.3)	0.018	38,924 (5.1)	61,883 (5.1)	<0.001
	Non-Hispanic	631,927 (83.4)	997,153 (82.8)		630,037 (83.0)	997,945 (83.0)	
	Unknown	87,920 (11.6)	144,483 (12.0)		89,842 (11.8)	142,383 (11.8)	
Region, N (%)	Midwest	153,932 (20.3)	287,611 (23.9)	0.089	172,470 (22.7)	272,229 (22.6)	0.002
	Northeast	194,796 (25.7)	306,893 (25.5)		193,511 (25.5)	307,058 (25.5)	
	South	229,485 (30.3)	339,329 (28.2)		218,508 (28.8)	346,982 (28.9)	
	West	151,598 (20.0)	229,463 (19.0)		147,450 (19.4)	233,374 (19.4)	
	Unknown	27,761 (3.7)	41,679 (3.5)		26,865 (3.5)	42,569 (3.5)	
Month of index, N (%)	August 2022	7 (<0.1)	14 (<0.1)	0.068	9 (<0.1)	13 (<0.1)	0.001
	September 2022	165,746 (21.9)	295,324 (24.5)		178,227 (23.5)	282,665 (23.5)	
	October 2022	262,769 (34.7)	417,242 (34.6)		262,976 (34.7)	416,425 (34.6)	
	November 2022	161,844 (21.4)	241,826 (20.1)		156,093 (20.6)	247,126 (20.6)	
	December 2022	102,636 (13.5)	152,770 (12.7)		98,845 (13.0)	156,509 (13.0)	
	January 2023	46,739 (6.2)	70,112 (5.8)		45,090 (5.9)	71,559 (6.0)	
	February 2023	17,831 (2.4)	27,687 (2.3)		17,563 (2.3)	27,915 (2.3)	
Primary-series COVID-19 vaccine, N (%)	Heterologous	64,763 (8.5)	133,740 (11.1)	0.175	79,300 (10.5)	123,200 (10.2)	0.007
	Homologous	226,626 (29.9)	272,401 (22.6)		190,119 (25.1)	302,057 (25.1)	
	Not reported	466,183 (61.5)	798,834 (66.3)		489,385 (64.5)	776,955 (64.6)	
Time since last COVID-19 monovalent vaccination, N (%)	≤90 days	12,342 (1.6)	15,007 (1.2)	0.231	10,456 (1.4)	16,657 (1.4)	0.002
	91–180 days	158,876 (21.0)	161,599 (13.4)		122,413 (16.1)	193,483 (16.1)	
	>180 days	443,211 (58.5)	725,100 (60.2)		452,371 (59.6)	717,402 (59.7)	
	Not reported	143,143 (18.9)	303,269 (25.2)		173,564 (22.9)	274,670 (22.8)	
Time since last COVID-19 infection, N (%)	≤120 days	32,609 (4.3)	52,761 (4.4)	0.030	33,104 (4.4)	52,418 (4.4)	<0.001
	121–180 days	17,269 (2.3)	27,424 (2.3)		17,371 (2.3)	27,457 (2.3)	
	>180 days	67,135 (8.9)	117,056 (9.7)		71,407 (9.4)	113,132 (9.4)	
	Not reported	640,559 (84.6)	1,007,734 (83.6)		636,921 (83.9)	1,009,204 (83.9)	
Underlying medical conditions, N (%)	Asthma	107,684 (14.2)	175,269 (14.5)	0.009	109,615 (14.4)	173,562 (14.4)	<0.001
	Cancer	99,704 (13.2)	151,325 (12.6)	0.018	96,642 (12.7)	153,428 (12.8)	<0.001
	Cerebrovascular disease	68,237 (9.0)	104,581 (8.7)	0.012	66,354 (8.7)	105,537 (8.8)	0.001
	Chronic kidney disease	106,577 (14.1)	164,005 (13.6)	0.013	103,660 (13.7)	165,086 (13.7)	0.002
	Chronic liver diseases	14,318 (1.9)	22,958 (1.9)	0.001	14,377 (1.9)	22,830 (1.9)	<0.001
	Chronic lung diseases ^a^	98,839 (13.0)	150,479 (12.5)	0.017	95,808 (12.6)	152,296 (12.7)	0.001
	Cystic fibrosis	286 (<0.1)	422 (<0.1)	0.001	272 (<0.1)	431 (<0.1)	<0.001
	Diabetes type 1 and 2	252,180 (33.3)	388,331 (32.2)	0.023	246,489 (32.5)	391,550 (32.6)	0.002
	Disabilities	82,976 (11.0)	141,091 (11.7)	0.024	87,274 (11.5)	137,767 (11.5)	0.001
	Heart conditions	169,131 (22.3)	256,250 (21.3)	0.026	163,349 (21.5)	259,743 (21.6)	0.002
	HIV	7327 (1.0)	11,394 (0.9)	0.002	7269 (1.0)	11,491 (1.0)	<0.001
	Mental health disorders	201,996 (26.7)	342,635 (28.4)	0.040	211,585 (27.9)	334,556 (27.8)	0.001
	Neurologic conditions	26,905 (3.6)	47,214 (3.9)	0.019	28,561 (3.8)	45,438 (3.8)	<0.001
	Obesity	255,376 (33.7)	410,845 (34.1)	0.008	257,461 (33.9)	408,164 (34.0)	<0.001
	Physical inactivity	1200 (0.2)	1941 (0.2)	<0.001	1223 (0.2)	1931 (0.2)	<0.001
	Primary Immunodeficiencies	15,989 (2.1)	25,055 (2.1)	0.002	15,724 (2.1)	25,043 (2.1)	<0.001
	Pregnancy ^b^	5072 (0.7)	9587 (0.8)	0.015	5741 (0.8)	9048 (0.8)	<0.001
	Smoking ^c^	153,460 (20.3)	244,998 (20.3)	0.002	153,751 (20.3)	243,993 (20.3)	<0.001
	Solid organ or hematopoietic cell transplantation	5078 (0.7)	8679 (0.7)	0.006	5287 (0.7)	8429 (0.7)	<0.001
	Tuberculosis	444 (0.1)	719 (0.1)	<0.001	447 (0.1)	711 (0.1)	<0.001
	Use of immunosuppressants	63,796 (8.4)	98,130 (8.1)	0.010	62,513 (8.2)	99,075 (8.2)	<0.001

IQR, interquartile range; SD, standard deviation; SMD, standardized mean difference; ^a^ except for asthma; ^b^ includes recent pregnancy; ^c^ includes current and former smoker.

**Table 3:** Number of individuals included in the subgroup analyses and duration of follow-up.

	Pre-Weighting, N (%)		Post Weighting, N (%)		Follow-Up Duration in Days, Median (IQR), Pre-Weighting	
	mRNA-1273.222	BNT162b2 Bivalent	mRNA-1273.222	BNT162b2 Bivalent	mRNA-1273.222	BNT162b2 Bivalent
	N = 757,572	N = 1,204,975	N = 758,803	N = 1,202,211	N = 757,572	N = 1,204,975
Diabetes	252,180 (33.3)	388,331 (32.2)	252,635 (33.3)	387,233 (32.2)	195 (140–223)	197 (142–226)
Cerebro- and cardiovasculardisease	204,933 (27.1)	311,148 (25.8)	205,364 (27.1)	310,143 (25.8)	198 (144–225)	201 (145–228)
Chronic lung disease	183,686 (24.2)	290,887 (24.1)	183,969 (24.2)	290,178 (24.1)	195 (139–224)	197 (141–226)
Immunocompromised	169,185 (22.3)	258,467 (21.4)	169,509 (22.3)	257,751 (21.4)	199 (148–226)	202 (150–229)
Chronic kidney disease	106,577 (14.1)	164,005 (13.6)	106,841 (14.1)	163,426 (13.6)	196 (140–224)	199 (141–226)

IQR, interquartile range.

**Table 4:** Unadjusted and adjusted hazard ratios (HRs) and 95% confidence intervals (CIs) for mRNA-1273.222 versus BNT162b2 Bivalent.

	Unadjusted HR ^a^, (95% CI)		Adjusted HR ^b^, (95% CI)	
	COVID-19-Related Hospitalization	COVID-19-Related Outpatient Encounter	COVID-19-Related Hospitalization	COVID-19-Related Outpatient Encounter
Overall	0.873 (0.830–0.919)	0.971 (0.956–0.986)	0.891 (0.848–0.938)	0.968 (0.953–0.983)
Subgroup analyses				
Diabetes	0.802 (0.746–0.863)	0.947 (0.923–0.972)	0.849 (0.790–0.913)	0.963 (0.938–0.988)
Cerebro- and cardiovascular disease	0.790 (0.739–0.844)	0.894 (0.871–0.919)	0.853 (0.799–0.910)	0.926 (0.902–0.951)
Chronic lung disease	0.852 (0.791–0.918)	0.957 (0.932–0.984)	0.881 (0.818–0.949)	0.969 (0.943–0.996)
Immunocompromised	0.835 (0.764–0.912)	0.946 (0.917–0.976)	0.850 (0.778–0.928)	0.952 (0.923–0.982)
Chronic kidney disease	0.843 (0.775–0.916)	0.894 (0.860–0.928)	0.916 (0.843–0.995)	0.924 (0.890–0.959)
Sensitivity analyses				
Open claims	0.859 (0.833–0.887)	0.928 (0.919–0.937)	0.880 (0.853–0.907)	0.869 (0.818–0.922)
Closed claims—cut-off 28 February 2023	0.854 (0.804–0.907)	0.974 (0.957–0.991)	0.950 (0.941–0.960)	0.962 (0.946–0.979)

^a^ Pre-weighting, ^b^ post weighting.

**Table 5:** Adjusted relative vaccine effectiveness (rVE) estimates ^a^, sensitivity analyses.

Outcome	Adjusted rVE
Open claims—cut-off 31 May 2023	
COVID-19-related hospitalization	12.0% (9.3%–14.7%)
COVID-19-related outpatient encounter	5.0% (4.0%–5.9%)
Closed claims—cut-off 28 February 2023	
COVID-19-related hospitalization	13.1% (7.8%–18.2%)
COVID-19-related outpatient encounter	3.8% (2.1%–5.4%)

^a^ mRNA-1273.222 versus BNT162b2 Bivalent.

## Data Availability

The data that support the findings of this study were used under license from Veradigm and Komodo Health. Due to data use agreements and their proprietary nature, restrictions apply regarding the availability of the data. Further information is available from the corresponding author.

## Supplementary Materials

The following supporting information can be downloaded at https://www.mdpi.com/article/10.3390/vaccines12101107/s1, Table S1. Code list for underlying medical conditions. Table S2. Baseline characteristics of the diabetic cohort. Table S3. Baseline characteristics the cerebro- and cardiovascular disease cohort. Table S4. Baseline characteristics of the chronic lung disease cohort. Table S5. Baseline characteristics of the immunocompromised cohort. Table S6. Baseline characteristics of the chronic kidney disease cohort. Table S7. Unweighted, unadjusted relative vaccine effectiveness (rVE) estimates. Table S8. Baseline characteristics of individuals included in the open claims sensitivity analysis. Table S9. Baseline characteristics of individuals included in the sensitivity analysis using an end date of 28 February 2023.

## References

[1] Lythgoe K.A., Golubchik T., Hall M., House T., Cahuantzi R., MacIntyre-Cockett G., Fryer H., Thomson L., Nurtay A., Ghafani M. (2023). Lineage Replacement and Evolution Captured by 3 Years of the United Kingdom Coronavirus (COVID-19) Infection Survey. Proc. Biol. Sci..

[2] Chatterjee S., Bhattacharya M., Nag S., Dhama K., Chakraborty C. (2023). A Detailed Overview of SARS-CoV-2 Omicron: Its Sub-Variants, Mutations and Pathophysiology, Clinical Characteristics, Immunological Landscape, Immune Escape, and Therapies. Viruses.

[3] Tian D., Sun Y., Xu H., Ye Q. (2022). The Emergence and Epidemic Characteristics of the Highly Mutated SARS-CoV-2 Omicron Variant. J. Med. Virol..

[4] Chalkias S., Harper C., Vrbicky K., Walsh S.R., Essink B., Brosz A., McGhee N., Tomassini J.E., Chen X., Chang Y. (2022). A Bivalent Omicron-Containing Booster Vaccine against COVID-19. N. Engl. J. Med..

[5] Zou J., Kurhade C., Patel S., Kitchin N., Tompkins K., Cutler M., Cooper D., Yang Q., Cai H., Muik A. (2022). Improved Neutralization of Micron BA.4/5, BA.4.6, BA.2.75.2, BQ.1.1, and XBB.1 with Bivalent BA.4/5 Vaccine. BioRxiv.

[6] Rosenblum H.G. (2022). Interim Recommendations from the Advisory Committee on Immunization Practices for the Use of Bivalent Booster Doses of COVID-19 Vaccines—United States, October 2022. MMWR Morb. Mortal. Wkly. Rep..

[7] Kim L., Garg S., O’Halloran A., Whitaker M., Pham H., Anderson E.J., Armistead I., Bennett N.M., Billing L., Como-Sabetti K. (2021). Risk Factors for Intensive Care Unit Admission and In-Hospital Mortality among Hospitalized Adults Identified through the US Coronavirus Disease 2019 (COVID-19)-Associated Hospitalization Surveillance Network (COVID-NET). Clin. Infect. Dis..

[8] Rosenthal N., Cao Z., Gundrum J., Sianis J., Safo S. (2020). Risk Factors Associated with In-Hospital Mortality in a US National Sample of Patients with COVID-19. JAMA Netw. Open.

[9] Boersma P., Black L.I., Ward B.W. (2020). Prevalence of Multiple Chronic Conditions among US Adults, 2018. Prev. Chronic Dis..

[10] Kopel H., Bogdanov A., Winer-Jones J.P., Adams C., Winer I.H., Bonafede M., Nguyen V.H., Mansi J.A. (2024). Comparison of COVID-19 and Influenza-Related Outcomes in the United States during Fall–Winter 2022–2023: A Cross-Sectional Retrospective Study. Diseases.

[11] Centers for Disease Control and Prevention COVID-NET: COVID-19-Associated Hospitalization Surveillance Network. https://www.cdc.gov/coronavirus/2019-ncov/covidnetdashboard/de/powerbi/dashboard.html.

[12] Kopel H., Nguyen V.H., Boileau C., Bogdanov A., Winer I., Ducruet T., Zeng N., Bonafede M., Esposito D.B., Martin D. (2023). Comparative Effectiveness of Bivalent (Original/Omicron BA.4/BA.5) COVID-19 Vaccines in Adults. Vaccines.

[13] Boikos C., McGovern I., Molrine D., Ortiz J.R., Puig-Barberà J., Haag M. (2022). Review of Analyses Estimating Relative Vaccine Effectiveness of Cell-Based Quadrivalent Influenza Vaccine in Three Consecutive US Influenza Seasons. Vaccines.

[14] Nguyen V.H., Boileau C., Bogdanov A., Sredl M., Bonafede M., Ducruet T., Chavers S., Rosen A., Martin D., Buck P. (2023). Relative Effectiveness of BNT162b2, mRNA-1273, and Ad26.COV2.S Vaccines and Homologous Boosting in Preventing COVID-19 in Adults in the US. Open Forum Infect. Dis..

[15] Centers for Disease Control and Prevention Underlying Medical Conditions Associated with Higher Risk for Severe COVID-19: Information for Healthcare Professionals. https://www.cdc.gov/covid/hcp/clinical-care/underlying-conditions.html.

[16] Benavidez G.A., Zahnd W.E., Hung P., Eberth J.M. (2024). Chronic Disease Prevalence in the US: Sociodemographic and Geographic Variations by Zip Code Tabulation Area. Prev. Chronic Dis..

[17] Hulme W.J., Horne E.M.F., Parker E.P.K., Keogh R.H., Williamson E.J., Walker V., Palmer T.M., Curtis H.J., Walker A.J., Andrews C.D. (2023). Comparative Effectiveness of BNT162b2 versus mRNA-1273 Covid-19 Vaccine Boosting in England: Matched Cohort Study in OpenSAFELY-TPP. BMJ.

[18] Dickerman B.A., Gerlovin H., Madenci A.L., Figueroa Muñiz M.J., Wise J.K., Adhikari N., Ferolito B.R., Kurgansky K.E., Gagnon D.R., Cho K. (2023). Comparative Effectiveness of Third Doses of mRNA-Based COVID-19 Vaccines in US Veterans. Nat. Microbiol..

[19] Embi P.J., Levy M.E., Naleway A.L., Patel P., Gaglani M., Natarajan K., Dascomb K., Ong T.C., Klein N.P., Liao I.-C. (2021). Effectiveness of 2-Dose Vaccination with mRNA COVID-19 Vaccines Against COVID-19-Associated Hospitalizations Among Immunocompromised Adults - Nine States, January-September 2021. MMWR Morb Mortal Wkly Rep..

[20] Drawz P.E., DeSilva M., Bodurtha P., Vazquez Benitez G., Murray A., Chamberlain A.M., Dudley R.A., Waring S., Kharbanda A.B., Murphy D. (2022). Effectiveness of BNT162b2 and mRNA-1273 Second Doses and Boosters for Severe Acute Respiratory Syndrome Coronavirus 2 (SARS-CoV-2) Infection and SARS-CoV-2-Related Hospitalizations: A Statewide Report from the Minnesota Electronic Health Record Consortium. Clin. Infect. Dis..

[21] Molnár G.A., Vokó Z., Sütő G., Rokszin G., Nagy D., Surján G., Surján O., Nagy P., Kenessey I., Wéber A. (2024). Effectiveness of SARS-CoV-2 Primary Vaccines and Boosters in Patients with Type 2 Diabetes Mellitus in Hungary (HUN-VE 4 Study). BMJ Open Diabetes Res. Care.

[22] Kavikondala S., Haeussler K., Wang X., Spellman A., Bausch-Jurken M.T., Sharma P., Amiri M., Krivelyova A., Vats S., Nassim M. (2024). Immunogenicity of mRNA-1273 and BNT162b2 in Immunocompromised Patients: Systematic Review and Meta-Analysis Using GRADE. Infect. Dis. Ther..

[23] Mavrovouniotis I., Fylaktou A., Stagou M., Ouranos K., Lioulios G., Evgenikaki E., Exindari M., Gioula G. (2023). Cellular and Humoral Responses in Dialysis Patients after Vaccination with the BNT162b2 or mRNA-1273 Vaccines. Life.

[24] Liao S.-Y., Gerber A.N., Zelarney P., Make B., Wechsler M.E. (2022). Impaired SARS-CoV-2 mRNA Vaccine Antibody Response in Chronic Medical Conditions: A Real-World Analysis. Chest.

[25] Jia X., Dong F., Pyle L., Michels A.W., Yu L., Rewers M. (2023). Similar Time Course of Humoral Response to SARS-CoV-2 mRNA Vaccines in People with and without Type 1 Diabetes. Diabetes Technol. Ther..

[26] Neuhann J.M., Stemler J., Carcas A.J., Frías-Iniesta J., Akova M., Bethe U., Heringer S., Salmanton-García J., Tischmann L., Zarrouk M. (2023). Immunogenicity and Reactogenicity of a First Booster with BNT162b2 or Full-Dose mRNA-1273: A Randomised VACCELERATE Trial in Adults ≥75 Years (EU-COVAT-1). Vaccine.

[27] Clark A., Jit M., Warren-Gash C., Guthrie B., Wang H.H.X., Mercer S.W., Sanderson C., McKee M., Troeger C., Ong K.L. (2020). Global, Regional, and National Estimates of the Population at Increased Risk of Severe COVID-19 Due to Underlying Health Conditions in 2020: A Modelling Study. Lancet Glob. Health.

[28] Fried M.W., Crawford J.M., Mospan A.R., Watkins S.E., Munoz B., Zink R.C., Elliott S., Burleson K., Landis C., Reddy K.R. (2021). Patient Characteristics and Outcomes of 11 721 Patients with Coronavirus Disease 2019 (COVID-19) Hospitalized across the United States. Clin. Infect. Dis..

[29] Harrison S.L., Fazio-Eynullayeva E., Lane D.A., Underhill P., Lip G.Y.H. (2020). Comorbidities Associated with Mortality in 31,461 Adults with COVID-19 in the United States: A Federated Electronic Medical Record Analysis. PLoS Med..

[30] Gallo Marin B., Aghagoli G., Lavine K., Yang L., Siff E.J., Chiang S.S., Salazar-Mather T.P., Dumenco L., Savaria M.C., Aung S.N. (2021). Predictors of COVID-19 Severity: A Literature Review. Rev. Med. Virol..

[31] Kompaniyets L. (2021). Underlying Medical Conditions and Severe Illness among 540,667 Adults Hospitalized with COVID-19, March 2020–March 2021. Prev. Chronic Dis..

[32] Shrestha S.S., Kompaniyets L., Grosse S.D., Harris A.M., Baggs J., Sircar K., Gundlapalli A.V. (2021). Estimation of Coronavirus Disease 2019 Hospitalization Costs from a Large Electronic Administrative Discharge Database, March 2020–July 2021. Open Forum Infect. Dis..

[33] Scott A., Ansari W., Khan F., Chambers R., Benigno M., Di Fusco M., McGrath L., Malhotra D., Draica F., Nguyen J. (2024). Substantial Health and Economic Burden of COVID-19 during the Year after Acute Illness among US Adults at High Risk of Severe COVID-19. BMC Med..

