# Supplementary material for: Comparative Effectiveness of the Bivalent (Original/Omicron BA.4/BA.5) mRNA COVID-19 Vaccines mRNA-1273.222 and BNT162b2 Bivalent in Adults with Underlying Medical Conditions in the United States
Source: Vaccines (Basel). 2024 Sep 27;12(10):1107. doi: 10.3390/vaccines12101107 (PMC11511346; doi:10.3390/vaccines12101107)
Supplement: Supplementary file 1 [file vaccines-12-01107-s001.zip › Supplementary files.pdf]

Supplementary files for “Comparative Effectiveness of the Bivalent (Original/Omicron BA.4/BA.5) mRNA COVID-19 Vaccines mRNA-1273.222 and BNT162b2 Bivalent in Adults With Underlying Medical Conditions in the United States”

Supplementary Tables

Supplementary Table S1. Code list for underlying medical conditions.

Supplementary Table S2. Baseline characteristics of the diabetic cohort. Data are presented as n (%) unless otherwise stated.

Supplementary Table S3. Baseline characteristics the cerebro- and cardiovascular cohort. Data are presented as n (%) unless otherwise stated

Supplementary Table S4. Baseline characteristics of the chronic lung disease cohort. Data are presented as n (%) unless otherwise stated

Supplementary Table S5. Baseline characteristics of the immunocompromised cohort. Data are presented as n (%) unless otherwise stated

Supplementary Table S6. Baseline characteristics of the chronic kidney disease cohort. Data are presented as n (%) unless otherwise stated

Supplementary Table S7: Unweighted, unadjusted relative vaccine effectiveness (rVE) estimates

	**COVID-19-related hospitalization**	**COVID-19-related outpatient encounter**
	rVE (95% CI)	rVE (95% CI)
Overall	12.7% (8.1%-17.0%)	2.9% (1.4%-4.4%)
Subgroup analyses		
Diabetes	19.8% (13.7%-25.4%)	5.3% (2.8%-7.7%)
Cerebro- and cardiovascular disease	21.0% (15.6%-26.1%)	10.6% (8.1%-12.9%)
Chronic lung disease	14.8% (8.2%-20.9%)	4.3% (1.6%-6.8%)
Immunocompromised	16.5% (8.8%-23.6%)	5.4% (2.4%-8.3%)
Chronic kidney disease	15.7% (8.4%-22.5%)	10.6% (7.2%-14.0%)
Sensitivity analyses		
Open claims	14.1% (11.3%-16.7%)	7.2% (6.3%-8.1%)
Closed claims – cut-off 28 Feb 23	14.6% (9.3%-19.6%)	2.6% (0.9%-4.3%)

Supplementary Table S8. Baseline characteristics of individuals included in the open claims sensitivity analysis. Data are presented as n (%) unless otherwise stated

Supplementary Table S9. Baseline characteristics of individuals included in the sensitivity analysis using an end date of February 28, 2023. Data are presented as n (%) unless otherwise stated

Supplementary Table S1. Code list for underlying medical conditions.

Condition	Type	Codes
Asthma	ICD-10-CM Dx	J45, J452, J4520, J4521, J4522, J4530, J4531, J4532, J454, J4540, J4541, J4542, J455, J4550, J4551, J459, J45901, J45902, J45909, J45990, J45991, J45998
Cancer	ICD-10-CM Dx	C00, C000, C001, C002, C003, C004, C005, C006, C008, C009, C01, C021, C022, C023, C024, C028, C029, C03, C030, C031, C039, C04, C040, C041, C048, C049, C05, C050, C051, C052, C058, C059, C06, C060, C061, C062, C068, C0680, C0689, C069, C07, C08, C080, C081, C089, C09, C090, C091, C098, C099, C10, C100, C101, C102, C103, C104, C108, C109, C11, C110, C111, C112, C113, C118, C119, C12, C13, C130, C131, C132, C138, C139, C14, C140, C142, C148, C15, C153, C154, C155, C158, C159, C16, C160, C161, C162, C163, C164, C165, C166, C168, C169, C17, C170, C171, C172, C173, C178, C179, C18, C180, C181, C182, C183, C184, C185, C186, C187, C188, C189, C19, C20, C21, C210, C211, C212, C218, C22, C220, C221, C222, C223, C224, C227, C228, C229, C23, C24, C240, C241, C248, C249, C25, C250, C251, C252, C253, C254, C257, C258, C259, C26, C260, C261, C269, C30, C300, C301, C31, C310, C311, C312, C313, C318, C319, C32, C320, C321, C322, C323, C328, C329, C33, C34, C340, C3400, C3401, C3402, C341, C3410, C3411, C3412, C342, C343, C3430, C3431, C3432, C348, C3480, C3481, C3482, C349, C3490, C3491, C3492, C37, C38, C380, C381, C382, C383, C384, C388, C39, C390, C399, C40, C400, C4000, C4001, C4002, C401, C4010, C4011, C4012, C402, C4020, C4021, C4022, C403, C4030, C4031, C4032, C408, C4080, C4081, C4082, C409, C4090, C4091, C4092, C41, C410, C411, C412, C413, C414, C419, C43, C430, C431, C4310, C4311, C43111, C43112, C4312, C43121, C43122, C432, C4320, C4321, C4322, C433, C4330, C4331, C4339, C434, C435, C4351, C4352, C4359, C436, C4360, C4361, C4362, C437, C4370, C4371, C4372, C438, C439, C45, C450, C451, C452, C457, C459, C46, C460, C461, C462, C463, C464, C465, C4650, C4651, C4652, C467, C469, C47, C470, C471, C4710, C4711, C4712, C472, C4720, C4721, C4722, C473, C474, C475, C476, C478, C479, C48, C480, C481, C482, C488, C49, C490, C491, C4910, C4911, C4912, C492, C4920, C4921, C4922, C493, C494, C495, C496, C498, C499, C49A, C49A0, C49A1, C49A2, C49A3, C49A4, C49A5, C49A9, C50, C500, C5001, C50011, C50012, C50019, C5002, C50021, C50022, C50029, C501, C5011, C50111, C50112, C50119, C5012, C50121, C50122, C50129, C502, C5021, C50211, C50212, C50219, C5022, C50221, C50222, C50229, C503, C5031, C50311, C50312, C50319, C5032, C50321, C50322, C50329, C504, C5041, C50411, C50412, C50419, C5042, C50421, C50422, C50429, C505, C5051, C50511, C50512, C50519, C5052, C50521, C50522, C50529, C506, C5061, C50611, C50612, C50619, C5062, C50621, C50622, C50629, C508, C5081, C50811, C50812, C50819, C5082, C50821, C50822, C50829, C509, C5091, C50911, C50912, C50919, C5092, C50921, C50922, C50929, C51, C510, C511, C512, C518, C519, C52, C53, C530, C531, C538, C539, C54, C540, C541, C542, C543, C548, C549, C55, C56, C561, C562, C569, C57, C570, C5700, C5701, C5702, C571, C5710, C5711, C5712, C572, C5720, C5721, C5722, C573, C574, C577, C578, C579, C58, C60, C600, C601, C602, C608, C609, C61, C62, C620, C6200, C6201, C6202, C621, C6210, C6211, C6212, C629, C6290, C6291, C6292, C63, C630, C6300, C6301, C6302, C631, C6310, C6311, C6312, C632, C637, C638, C639, C64, C641, C642, C649, C65, C651, C652, C659, C66, C661, C662, C669, C67, C670, C671, C672, C673, C674, C675, C676, C677, C678, C679, C68, C680, C681, C688, C689, C69, C690, C6900, C6901, C6902, C691, C6910, C6911, C6912, C692, C6920, C6921, C6922, C693, C6930, C6931, C6932, C694, C6940, C6941, C6942, C695, C6950, C6951, C6952, C696, C6960, C6961, C6962, C698, C6980, C6981, C6982, C699, C6990, C6991, C6992, C70, C700, C701, C709, C71, C710, C711, C712, C713, C714, C715, C716, C717, C718, C719, C72, C720, C721, C722, C7220, C7221, C7222, C723, C7230, C7231, C7232, C724, C7240, C7241, C7242, C725, C7250, C7259, C729, C73, C74, C740, C7400, C7401, C7402, C741, C7410, C7411, C7412, C749, C7490, C7491, C7492, C75, C750, C751, C752, C753, C754, C755, C758, C759, C76, C760, C761, C762, C763, C764, C7640, C7641, C7642, C765, C7650,

		C7651, C7652, C768, C81, C810, C8100, C8101, C8102, C8103, C8104, C8105, C8106, C8107, C8108, C8109, C811, C8110, C8111, C8112, C8113, C8114, C8115, C8116, C8117, C8118, C8119, C812, C8120, C8121, C8122, C8123, C8124, C8125, C8126, C8127, C8128, C8129, C813, C8130, C8131, C8132, C8133, C8134, C8135, C8136, C8137, C8138, C8139, C814, C8140, C8141, C8142, C8143, C8144, C8145, C8146, C8147, C8148, C8149, C817, C8170, C8171, C8172, C8173, C8174, C8175, C8176, C8177, C8178, C8179, C819, C8190, C8191, C8192, C8193, C8194, C8195, C8196, C8197, C8198, C8199, C82, C820, C8200, C8201, C8202, C8203, C8204, C8205, C8206, C8207, C8208, C8209, C821, C8210, C8211, C8212, C8213, C8214, C8215, C8216, C8217, C8218, C8219, C822, C8220, C8221, C8222, C8223, C8224, C8225, C8226, C8227, C8228, C8229, C823, C8230, C8231, C8232, C8233, C8234, C8235, C8236, C8237, C8238, C8239, C824, C8240, C8241, C8242, C8243, C8244, C8245, C8246, C8247, C8248, C8249, C825, C8250, C8251, C8252, C8253, C8254, C8255, C8256, C8257, C8258, C8259, C826, C8260, C8261, C8262, C8263, C8264, C8265, C8266, C8267, C8268, C8269, C828, C8280, C8281, C8282, C8283, C8284, C8285, C8286, C8287, C8288, C8289, C829, C8290, C8291, C8292, C8293, C8294, C8295, C8296, C8297, C8298, C8299, C83, C830, C8300, C8301, C8302, C8303, C8304, C8305, C8306, C8307, C8308, C8309, C831, C8310, C8311, C8312, C8313, C8314, C8315, C8316, C8317, C8318, C8319, C833, C8330, C8331, C8332, C8333, C8334, C8335, C8336, C8337, C8338, C8339, C835, C8350, C8351, C8352, C8353, C8354, C8355, C8356, C8357, C8358, C8359, C837, C8370, C8371, C8372, C8373, C8374, C8375, C8376, C8377, C8378, C8379, C838, C8380, C8381, C8382, C8383, C8384, C8385, C8386, C8387, C8388, C8389, C839, C8390, C8391, C8392, C8393, C8394, C8395, C8396, C8397, C8398, C8399, C84, C840, C8400, C8401, C8402, C8403, C8404, C8405, C8406, C8407, C8408, C8409, C841, C8410, C8411, C8412, C8413, C8414, C8415, C8416, C8417, C8418, C8419, C844, C8440, C8441, C8442, C8443, C8444, C8445, C8446, C8447, C8448, C8449, C846, C8460, C8461, C8462, C8463, C8464, C8465, C8466, C8467, C8468, C8469, C847, C8470, C8471, C8472, C8473, C8474, C8475, C8476, C8477, C8478, C8479, C84A, C84A0, C84A1, C84A2, C84A3, C84A4, C84A5, C84A6, C84A7, C84A8, C84A9, C84Z, C84Z0, C84Z1, C84Z2, C84Z3, C84Z4, C84Z5, C84Z6, C84Z7, C84Z8, C84Z9, C849, C8490, C8491, C8492, C8493, C8494, C8495, C8496, C8497, C8498, C8499, C85, C851, C8510, C8511, C8512, C8513, C8514, C8515, C8516, C8517, C8518, C8519, C852, C8520, C8521, C8522, C8523, C8524, C8525, C8526, C8527, C8528, C8529, C858, C8580, C8581, C8582, C8583, C8584, C8585, C8586, C8587, C8588, C8589, C859, C8590, C8591, C8592, C8593, C8594, C8595, C8596, C8597, C8598, C8599, C88, C880, C882, C883, C884, C888, C889, C90, C900, C9000, C9001, C9002, C901, C9010, C9011, C9012, C9902, C9020, C9021, C9022, C903, C9030, C9031, C9032, C91, C910, C9100, C9101, C9102, C9110, C9111, C9112, C9130, C9131, C9132, C9140, C9141, C9142, C9150, C9151, C9152, C9160, C9161, C9162, C91A0, C91A1, C91A2, C91Z0, C91Z1, C91Z2, C9190, C9191, C9192, C92, C920, C9200, C9201, C9202, C921, C9210, C9211, C9212, C922, C9220, C9221, C9222, C923, C9230, C9231, C9232, C924, C9240, C9241, C9242, C925, C9250, C9251, C9252, C926, C9260, C9261, C9262, C92A, C92A0, C92A1, C92A2, C92Z, C92Z0, C92Z1, C92Z2, C929, C9290, C9291, C9292, C93, C930, C9300, C9301, C9302, C931, C9310, C9311, C9312, C933, C9330, C9331, C9332, C93Z, C93Z0, C93Z1, C93Z2, C939, C9390, C9391, C9392, C94, C940, C9400, C9401, C9402, C942, C9420, C9421, C9422, C943, C9430, C9431, C9432, C944, C9440, C9441, C9442, C946, C948, C9480, C9481, C9482, C95, C950, C9500, C9501, C9502, C951, C9510, C9511, C9512, C959, C9590, C9591, C9592, C96, C960, C962, C9620, C9621, C9622, C9629, C964, C965, C966, C96A, C96Z, C969, C77, C770, C771, C772, C773, C774, C775, C778, C779, C78, C780, C7800, C7801, C7802, C781, C782, C783, C7830, C7839, C784, C785, C786, C787, C788, C7880, C7889, C79, C790, C7900, C7901, C7902, C791, C7910, C7911, C7919, C792, C793, C7931, C7932, C794, C7940, C7949, C795, C7951, C7952, C796, C7960, C7961, C7962, C797, C7970, C7971, C7972, C798, C7981, C7982, C7989, C799, C80, C800, C801, C802

Cerebrovascular disease	ICD-10-CM Dx	I6000, I6001, I6002, I6010, I6011, I6012, I602, I6030, I6031, I6032, I604, I6050, I6051, I6052, I606, I607, I608, I609, I610, I611, I612, I613, I614, I615, I616, I618, I619, I6200, I6201, I6202, I6203, I621, I629, I6300, I63011, I63012, I63013, I63019, I6302, I63031, I63032, I63033, I63039, I6309, I6310, I63111, I63112, I63113, I63119, I6312, I63131, I63132, I63133, I63139, I6319, I6320, I63211, I63212, I63213, I63219, I6322, I63231, I63232, I63233, I63239, I6329, I6330, I63311, I63312, I63313, I63319, I63321, I63322, I63323, I63329, I63331, I63332, I63333, I63339, I63341, I63342, I63343, I63349, I6339, I6340, I63411, I63412, I63413, I63419, I63421, I63422, I63423, I63429, I63431, I63432, I63433, I63439, I63441, I63442, I63443, I63449, I6349, I6350, I63511, I63512, I63513, I63519, I63521, I63522, I63523, I63529, I63531, I63532, I63533, I63539, I63541, I63542, I63543, I63549, I6359, I636, I6381, I6389, I639, I6501, I6502, I6503, I6509, I651, I6521, I6522, I6523, I6529, I658, I659, I6601, I6602, I6603, I6609, I6611, I6612, I6613, I6619, I6621, I6622, I6623, I6629, I663, I668, I669, I670, I671, I672, I673, I674, I675, I676, I677, I6781, I6782, I6783, I67841, I67848, I67850, I67858, I6789, I679, I680, I682, I688, I6900, I69010, I69011, I69012, I69013, I69014, I69015, I69018, I69019, I69020, I69021, I69022, I69023, I69028, I69031, I69032, I69033, I69034, I69039, I69041, I69042, I69043, I69044, I69049, I69051, I69052, I69053, I69054, I69059, I69061, I69062, I69063, I69064, I69065, I69069, I69090, I69091, I69092, I69093, I69098, I6910, I69110, I69111, I69112, I69113, I69114, I69115, I69118, I69119, I69120, I69121, I69122, I69123, I69128, I69131, I69132, I69133, I69134, I69139, I69141, I69142, I69143, I69144, I69149, I69151, I69152, I69153, I69154, I69159, I69161, I69162, I69163, I69164, I69165, I69169, I69190, I69191, I69192, I69193, I69198, I6920, I69210, I69211, I69212, I69213, I69214, I69215, I69218, I69219, I69220, I69221, I69222, I69223, I69228, I69231, I69232, I69233, I69234, I69239, I69241, I69242, I69243, I69244, I69249, I69251, I69252, I69253, I69254, I69259, I69261, I69262, I69263, I69264, I69265, I69269, I69290, I69291, I69292, I69293, I69298, I6930, I69310, I69311, I69312, I69313, I69314, I69315, I69318, I69319, I69320, I69321, I69322, I69323, I69328, I69331, I69332, I69333, I69334, I69339, I69341, I69342, I69343, I69344, I69349, I69351, I69352, I69353, I69354, I69359, I69361, I69362, I69363, I69364, I69365, I69369, I69390, I69391, I69392, I69393, I69398, I6980, I69810, I69811, I69812, I69813, I69814, I69815, I69818, I69819, I69820, I69821, I69822, I69823, I69828, I69831, I69832, I69833, I69834, I69839, I69841, I69842, I69843, I69844, I69849, I69851, I69852, I69853, I69854, I69859, I69861, I69862, I69863, I69864, I69865, I69869, I69890, I69891, I69892, I69893, I69898, I6990, I69910, I69911, I69912, I69913, I69914, I69915, I69918, I69919, I69920, I69921, I69922, I69923, I69928, I69931, I69932, I69933, I69934, I69939, I69941, I69942, I69943, I69944, I69949, I69951, I69952, I69953, I69954, I69959, I69961, I69962, I69963, I69964, I69965, I69969, I69990, I69991, I69992, I69993, I69998
Chronic kidney disease	ICD-10-CM Dx	N181, N182, N1830, N1831, N1832, N184, N185, N186, N189
Chronic liver disease	ICD-10-CM Dx	K700, K7010, K7011, K702, K7030, K7031, K7040, K7041, K709, K743, K744, K745, K7460, K7469, K754, K7581, K7589
Chronic lung diseases (except asthma)	ICD-10-CM Dx	I2601, I2602, I2609, I2690, I2692, I2693, I2694, I2699, I2720, I2721, I2722, I2723, I2724, I2729, J440, J441, J449, J470, J471, J479, J80, J810, J811, J8281, J8282, J8283, J8289, J8401, J8402, J8403, J8409, J8410, J84111, J84112, J84113, J84114, J84115, J84116, J84117, J84170, J84178, J842, J8481, J8482, J8483, J84841, J84842, J84843, J84848, J8489, J849, P271
Cystic fibrosis	ICD-10-CM Dx	E84, E840, E841, E8411, E8419, E848, E849

Diabetes	ICD-10-CM Dx	E1010, E1011, E1021, E1022, E1029, E10311, E10319, E103211, E103212, E103213, E103219, E103291, E103292, E103293, E103299, E103311, E103312, E103313, E103319, E103391, E103392, E103393, E103399, E103411, E103412, E103413, E103419, E103491, E103492, E103493, E103499, E103511, E103512, E103513, E103519, E103521, E103522, E103523, E103529, E103531, E103532, E103533, E103539, E103541, E103542, E103543, E103549, E103551, E103552, E103553, E103559, E103591, E103592, E103593, E103599, E1036, E1037X1, E1037X2, E1037X3, E1037X9, E1039, E1040, E1041, E1042, E1043, E1044, E1049, E1051, E1052, E1059, E10610, E10618, E10620, E10621, E10622, E10628, E10630, E10638, E10641, E10649, E1065, E1069, E108, E109, E1100, E1101, E1110, E1111, E1121, E1122, E1129, E11311, E11319, E113211, E113212, E113213, E113219, E113291, E113292, E113293, E113299, E113311, E113312, E113313, E113319, E113391, E113392, E113393, E113399, E113411, E113412, E113413, E113419, E113491, E113492, E113493, E113499, E113511, E113512, E113513, E113519, E113521, E113522, E113523, E113529, E113531, E113532, E113533, E113539, E113541, E113542, E113543, E113549, E113551, E113552, E113553, E113559, E113591, E113592, E113593, E113599, E1136, E1137X1, E1137X2, E1137X3, E1137X9, E1139, E1140, E1141, E1142, E1143, E1144, E1149, E1151, E1152, E1159, E11610, E11618, E11620, E11621, E11622, E11628, E11630, E11638, E11641, E11649, E1165, E1169, E118, E119,
Diasabilities	ICD-10-CM Dx	F70, F71, F72, F73, F78, F78A, F78A1, F78A9, F79, F80, F800, F801, F802, F804, F808, F8081, F8082, F8089, F809, F81, F810, F812, F818, F8181, F8189, F819, F82, F84, F840, F842, F843, F845, F848, F849, F88, F89, F90, F900, F901, F902, F908, F909, G80, G800, G801, G802, G803, G804, G808, G809, Q00, Q000, Q001, Q002, Q01, Q010, Q011, Q012, Q018, Q019, Q02, Q03, Q030, Q031, Q038, Q039, Q04, Q040, Q041, Q042, Q043, Q044, Q045, Q046, Q048, Q049, Q05, Q050, Q051, Q052, Q053, Q054, Q055, Q056, Q057, Q058, Q059, Q06, Q060, Q061, Q062, Q063, Q064, Q068, Q069, Q07, Q070, Q0700, Q0701, Q0702, Q0703, Q078, Q079, Q10, Q100, Q101, Q102, Q103, Q104, Q105, Q106, Q107, Q11, Q110, Q111, Q112, Q113, Q12, Q120, Q121, Q122, Q123, Q124, Q128, Q129, Q13, Q130, Q131, Q132, Q133, Q134, Q135, Q138, Q1381, Q1389, Q139, Q14, Q140, Q141, Q142, Q143, Q148, Q149, Q15, Q150, Q158, Q159, Q16, Q160, Q161, Q162, Q163, Q164, Q165, Q169, Q17, Q170, Q171, Q172, Q173, Q174, Q175, Q178, Q179, Q18, Q180, Q181, Q182, Q183, Q184, Q185, Q186, Q187, Q188, Q189, Q20, Q200, Q201, Q202, Q203, Q204, Q205, Q206, Q208, Q209, Q21, Q210, Q211, Q212, Q213, Q214, Q218, Q219, Q22, Q220, Q221, Q222, Q223, Q224, Q225, Q226, Q228, Q229, Q23, Q230, Q231, Q232, Q233, Q234, Q238, Q239, Q24, Q240, Q241, Q242, Q243, Q244, Q245, Q246, Q248, Q249, Q25, Q250, Q251, Q252, Q2521, Q2529, Q253, Q254, Q2540, Q2541, Q2542, Q2543, Q2544, Q2545, Q2546, Q2547, Q2548, Q2549, Q255, Q256, Q257, Q2571, Q2572, Q2579, Q258, Q259, Q26, Q260, Q261, Q262, Q263, Q264, Q265, Q266, Q268, Q269, Q27, Q270, Q271, Q272, Q273, Q2730, Q2731, Q2732, Q2733, Q2734, Q2739, Q274, Q278, Q279, Q28, Q280, Q281, Q282, Q283, Q288, Q289, Q30, Q300, Q301, Q302, Q303, Q308, Q309, Q31, Q310, Q311, Q312, Q313, Q315, Q318, Q319, Q32, Q320, Q321, Q322, Q323, Q324, Q33, Q330, Q331, Q332, Q333, Q334, Q335, Q336, Q338, Q339, Q34, Q340, Q341, Q348, Q349, Q35, Q351, Q353, Q355, Q357, Q359, Q36, Q360, Q361, Q369, Q37, Q370, Q371, Q372, Q373, Q374, Q375, Q378, Q379, Q38, Q380, Q381, Q382, Q383, Q384, Q385, Q386, Q387, Q388, Q39, Q390, Q391, Q392, Q393, Q394, Q395, Q396, Q398, Q399, Q40, Q400, Q401, Q402, Q403, Q408, Q409, Q41, Q410, Q411, Q412, Q418, Q419, Q42, Q420, Q421, Q422, Q423, Q428, Q429, Q43, Q430, Q431, Q432, Q433, Q434, Q435, Q436, Q437, Q438, Q439, Q44, Q440, Q441, Q442, Q443, Q444, Q445, Q446, Q447, Q45, Q450, Q451, Q452, Q453, Q458, Q459, Q50, Q500, Q5001, Q5002, Q501, Q502, Q503, Q5031, Q5032, Q5039, Q504, Q505, Q506, Q51, Q510, Q511, Q5110, Q5111, Q512, Q5121, Q5122, Q5128, Q513, Q514, Q515, Q516, Q517, Q518, Q5181, Q51810, Q51811, Q51818, Q5182, Q51820, Q51821, Q51828, Q519, Q52, Q520, Q521, Q5210, Q5211, Q5212, Q52120, Q52121, Q52122, Q52123, Q52124, Q52129, Q522, Q523, Q524, Q525, Q526, Q527, Q5270, Q5271, Q5279, Q528, Q529, Q53, Q530, Q5300, Q5301, Q5302, Q531, Q5310, Q5311, Q53111, Q53112, Q5312, Q5313, Q532, Q5320, Q5321, Q53211, Q53212, Q5322, Q5323,

		Q539, Q54, Q540, Q541, Q542, Q543, Q544, Q548, Q549, Q55, Q550, Q551, Q552, Q5520, Q5521, Q5522, Q5523, Q5529, Q553, Q554, Q555, Q556, Q5561, Q5562, Q5563, Q5564, Q5569, Q557, Q558, Q559, Q56, Q560, Q561, Q562, Q563, Q564, Q60, Q600, Q601, Q602, Q603, Q604, Q605, Q606, Q61, Q610, Q6100, Q6101, Q6102, Q611, Q6111, Q6119, Q612, Q613, Q614, Q615, Q618, Q619, Q62, Q620, Q621, Q6210, Q6211, Q6212, Q622, Q623, Q6231, Q6232, Q6239, Q624, Q625, Q626, Q6260, Q6261, Q6262, Q6263, Q6269, Q627, Q628, Q63, Q630, Q631, Q632, Q633, Q638, Q639, Q64, Q640, Q641, Q6410, Q6411, Q6412, Q6419, Q642, Q643, Q6431, Q6432, Q6433, Q6439, Q644, Q645, Q646, Q647, Q6470, Q6471, Q6472, Q6473, Q6474, Q6475, Q6479, Q648, Q649, Q65, Q650, Q6500, Q6501, Q6502, Q651, Q652, Q653, Q6530, Q6531, Q6532, Q654, Q655, Q656, Q658, Q6581, Q6582, Q6589, Q659, Q66, Q660, Q6600, Q6601, Q6602, Q661, Q6610, Q6611, Q6612, Q662, Q6621, Q66211, Q66212, Q66219, Q6622, Q66221, Q66222, Q66229, Q663, Q6630, Q6631, Q6632, Q664, Q6640, Q6641, Q6642, Q665, Q6650, Q6651, Q6652, Q666, Q667, Q6670, Q6671, Q6672, Q668, Q6680, Q6681, Q6682, Q6689, Q669, Q6690, Q6691, Q6692, Q67, Q670, Q671, Q672, Q673, Q674, Q675, Q676, Q677, Q678, Q68, Q680, Q681, Q682, Q683, Q684, Q685, Q686, Q688, Q69, Q690, Q691, Q692, Q699, Q70, Q700, Q7000, Q7001, Q7002, Q7003, Q701, Q7010, Q7011, Q7012, Q7013, Q702, Q7020, Q7021, Q7022, Q7023, Q703, Q7030, Q7031, Q7032, Q7033, Q704, Q709, Q71, Q710, Q7100, Q7101, Q7102, Q7103, Q711, Q7110, Q7111, Q7112, Q7113, Q712, Q7120, Q7121, Q7122, Q7123, Q713, Q7130, Q7131, Q7132, Q7133, Q714, Q7140, Q7141, Q7142, Q7143, Q715, Q7150, Q7151, Q7152, Q7153, Q716, Q7160, Q7161, Q7162, Q7163, Q718, Q7181, Q71811, Q71812, Q71813, Q71819, Q7189, Q71891, Q71892, Q71893, Q71899, Q719, Q7190, Q7191, Q7192, Q7193, Q72, Q720, Q7200, Q7201, Q7202, Q7203, Q721, Q7210, Q7211, Q7212, Q7213, Q722, Q7220, Q7221, Q7222, Q7223, Q723, Q7230, Q7231, Q7232, Q7233, Q724, Q7240, Q7241, Q7242, Q7243, Q725, Q7250, Q7251, Q7252, Q7253, Q726, Q7260, Q7261, Q7262, Q7263, Q727, Q7270, Q7271, Q7272, Q7273, Q728, Q7281, Q72811, Q72812, Q72813, Q72819, Q7289, Q72891, Q72892, Q72893, Q72899, Q729, Q7290, Q7291, Q7292, Q7293, Q73, Q730, Q731, Q738, Q74, Q740, Q741, Q742, Q743, Q748, Q749, Q75, Q750, Q751, Q752, Q753, Q754, Q755, Q758, Q759, Q76, Q760, Q761, Q762, Q763, Q764, Q7641, Q76411, Q76412, Q76413, Q76414, Q76415, Q76419, Q7642, Q76425, Q76426, Q76427, Q76428, Q76429, Q7649, Q765, Q766, Q767, Q768, Q769, Q77, Q770, Q771, Q772, Q773, Q774, Q775, Q776, Q777, Q778, Q779, Q78, Q780, Q781, Q782, Q783, Q784, Q785, Q786, Q788, Q789, Q79, Q790, Q791, Q792, Q793, Q794, Q795, Q7951, Q7959, Q796, Q7960, Q7961, Q7962, Q7963, Q7969, Q798, Q799, Q80, Q800, Q801, Q802, Q803, Q804, Q808, Q809, Q81, Q810, Q811, Q812, Q818, Q819, Q82, Q820, Q821, Q822, Q823, Q824, Q825, Q826, Q828, Q829, Q83, Q830, Q831, Q832, Q833, Q838, Q839, Q84, Q840, Q841, Q842, Q843, Q844, Q845, Q846, Q848, Q849, Q85, Q850, Q8500, Q8501, Q8502, Q8503, Q8509, Q851, Q858, Q859, Q86, Q860, Q861, Q862, Q868, Q87, Q870, Q871, Q8711, Q8719, Q872, Q873, Q874, Q8740, Q8741, Q87410, Q87418, Q8742, Q8743, Q875, Q878, Q8781, Q8782, Q8789, Q89, Q890, Q8901, Q8909, Q891, Q892, Q893, Q894, Q897, Q898, Q899, S14, S140, S140XXA, S140XXD, S140XXS, S141, S1410, S14101, S14101A, S14101D, S14101S, S14102, S14102A, S14102D, S14102S, S14103, S14103A, S14103D, S14103S, S14104, S14104A, S14104D, S14104S, S14105, S14105A, S14105D, S14105S, S14106, S14106A, S14106D, S14106S, S14107, S14107A, S14107D, S14107S, S14108, S14108A, S14108D, S14108S, S14109, S14109A, S14109D, S14109S, S1411, S14111, S14111A, S14111D, S14111S, S14112, S14112A, S14112D, S14112S, S14113, S14113A, S14113D, S14113S, S14114, S14114A, S14114D, S14114S, S14115, S14115A, S14115D, S14115S, S14116, S14116A, S14116D, S14116S, S14117, S14117A, S14117D, S14117S, S14118, S14118A, S14118D, S14118S, S14119, S14119A, S14119D, S14119S, S1412, S14121, S14121A, S14121D, S14121S, S14122, S14122A, S14122D, S14122S, S14123, S14123A, S14123D, S14123S, S14124, S14124A, S14124D, S14124S, S14125, S14125A, S14125D, S14125S, S14126, S14126A, S14126D, S14126S, S14127, S14127A, S14127D, S14127S, S14128, S14128A, S14128D, S14128S, S14129, S14129A, S14129D, S14129S, S1413, S14131, S14131A, S14131D, S14131S, S14132, S14132A, S14132D, S14132S, S14133, S14133A, S14133D, S14133S, S14134, S14134A, S14134D, S14134S, S14135,

		S14135A, S14135D, S14135S, S14136, S14136A, S14136D, S14136S, S14137, S14137A, S14137D, S14137S, S14138, S14138A, S14138D, S14138S, S14139, S14139A, S14139D, S14139S, S1414, S14141, S14141A, S14141D, S14141S, S14142, S14142A, S14142D, S14142S, S14143, S14143A, S14143D, S14143S, S14144, S14144A, S14144D, S14144S, S14145, S14145A, S14145D, S14145S, S14146, S14146A, S14146D, S14146S, S14147, S14147A, S14147D, S14147S, S14148, S14148A, S14148D, S14148S, S14149, S14149A, S14149D, S14149S, S1415, S14151, S14151A, S14151D, S14151S, S14152, S14152A, S14152D, S14152S, S14153, S14153A, S14153D, S14153S, S14154, S14154A, S14154D, S14154S, S14155, S14155A, S14155D, S14155S, S14156, S14156A, S14156D, S14156S, S14157, S14157A, S14157D, S14157S, S14158, S14158A, S14158D, S14158S, S14159, S14159A, S14159D, S14159S, S142, S142XXA, S142XXD, S142XXS, S143, S143XXA, S143XXD, S143XXS, S144, S144XXA, S144XXD, S144XXS, S145, S145XXA, S145XXD, S145XXS, S148, S148XXA, S148XXD, S148XXS, S149, S149XXA, S149XXD, S149XXS, S24, S240, S240XXA, S240XXD, S240XXS, S241, S2410, S24101, S24101A, S24101D, S24101S, S24102, S24102A, S24102D, S24102S, S24103, S24103A, S24103D, S24103S, S24104, S24104A, S24104D, S24104S, S24109, S24109A, S24109D, S24109S, S2411, S24111, S24111A, S24111D, S24111S, S24112, S24112A, S24112D, S24112S, S24113, S24113A, S24113D, S24113S, S24114, S24114A, S24114D, S24114S, S24119, S24119A, S24119D, S24119S, S2413, S24131, S24131A, S24131D, S24131S, S24132, S24132A, S24132D, S24132S, S24133, S24133A, S24133D, S24133S, S24134, S24134A, S24134D, S24134S, S24139, S24139A, S24139D, S24139S, S2414, S24141, S24141A, S24141D, S24141S, S24142, S24142A, S24142D, S24142S, S24143, S24143A, S24143D, S24143S, S24144, S24144A, S24144D, S24144S, S24149, S24149A, S24149D, S24149S, S2415, S24151, S24151A, S24151D, S24151S, S24152, S24152A, S24152D, S24152S, S24153, S24153A, S24153D, S24153S, S24154, S24154A, S24154D, S24154S, S24159, S24159A, S24159D, S24159S, S242, S242XXA, S242XXD, S242XXS, S243, S243XXA, S243XXD, S243XXS, S244, S244XXA, S244XXD, S244XXS, S248, S248XXA, S248XXD, S248XXS, S249, S249XXA, S249XXD, S249XXS, S34, S340, S3401, S3401XA, S3401XD, S3401XS, S3402, S3402XA, S3402XD, S3402XS, S341, S3410, S34101, S34101A, S34101D, S34101S, S34102, S34102A, S34102D, S34102S, S34103, S34103A, S34103D, S34103S, S34104, S34104A, S34104D, S34104S, S34105, S34105A, S34105D, S34105S, S34109, S34109A, S34109D, S34109S, S3411, S34111, S34111A, S34111D, S34111S, S34112, S34112A, S34112D, S34112S, S34113, S34113A, S34113D, S34113S, S34114, S34114A, S34114D, S34114S, S34115, S34115A, S34115D, S34115S, S34119, S34119A, S34119D, S34119S, S3412, S34121, S34121A, S34121D, S34121S, S34122, S34122A, S34122D, S34122S, S34123, S34123A, S34123D, S34123S, S34124, S34124A, S34124D, S34124S, S34125, S34125A, S34125D, S34125S, S34129, S34129A, S34129D, S34129S, S3413, S34131, S34131A, S34131D, S34131S, S34132, S34132A, S34132D, S34132S, S34139, S34139A, S34139D, S34139S, S342, S3421, S3421XA, S3421XD, S3421XS, S3422, S3422XA, S3422XD, S3422XS, S343, S343XXA, S343XXD, S343XXS, S344, S344XXA, S344XXD, S344XXS, S345, S345XXA, S345XXD, S345XXS, S346, S346XXA, S346XXD, S346XXS, S348, S348XXA, S348XXD, S348XXS
Heart disease	ICD-10-CM Dx	I200, I201, I208, I209, I2101, I2102, I2109, I2111, I2119, I2121, I2129, I213, I214, I219, I21A1, I21A9, I220, I221, I222, I228, I229, I230, I231, I232, I233, I234, I235, I236, I237, I238, I240, I241, I248, I249, I2510, I25110, I25111, I25118, I25119, I252, I253, I2541, I2542, I255, I256, I25700, I25701, I25708, I25709, I25710, I25711, I25718, I25719, I25720, I25721, I25728, I25729, I25730, I25731, I25738, I25739, I25750, I25751, I25758, I25759, I25760, I25761, I25768, I25769, I25790, I25791, I25798, I25799, I25810, I25811, I25812, I2582, I2583, I2584, I2589, I259, I420, I421, I422, I423, I424, I425, I426, I427, I428, I429, I501, I5020, I5021, I5022, I5023, I5030, I5031, I5032, I5033, I5040, I5041, I5042, I5043, I50810, I50811, I50812, I50813, I50814, I5082, I5083, I5084, I5089, I509
HIV	ICD-10-CM Dx	B20, B9735

Mental health disorder	ICD-10-CM Dx	F200, F201, F202, F203, F205, F2081, F2089, F209, F21, F22, F23, F24, F250, F251, F258, F259, F28, F29, F3010, F3011, F3012, F3013, F302, F303, F304, F308, F309, F310, F3110, F3111, F3112, F3113, F312, F3130, F3131, F3132, F314, F315, F3160, F3161, F3162, F3163, F3164, F3170, F3171, F3172, F3173, F3174, F3175, F3176, F3177, F3178, F3181, F3189, F319, F320, F321, F322, F323, F324, F325, F3281, F3289, F329, F330, F331, F332, F333, F3340, F3341, F3342, F338, F339, F340, F341, F3481, F3489, F349, F39
Neurological conditions limited to dementia	ICD-10-CM Dx	F01, F015, F0150, F0151, F02, F028, F0280, F0281, F03, F039, F0390, F0391, F04, F05, F061, F068, G132, G138, G30, G300, G301, G308, G309, G310, G3101, G3109, G311, G312, G914, G94, R4181, R54
Obesity	ICD-10-CM Dx	E6601, E6609, E661, E662, E668, E669, Z6830, Z6831, Z6832, Z6833, Z6834, Z6835, Z6836, Z6837, Z6838, Z6839, Z6841, Z6842, Z6843, Z6844, Z6845
Physical inactivity	ICD-10-CM Dx	Z723
Pregnancy	ICD-10-CM Dx	Z3A00, Z3A01, Z3A08, Z3A09, Z3A10, Z3A11, Z3A12, Z3A13, Z3A14, Z3A15, Z3A16, Z3A17, Z3A18, Z3A19, Z3A20, Z3A21, Z3A22, Z3A23, Z3A24, Z3A25, Z3A26, Z3A27, Z3A28, Z3A29, Z3A30, Z3A31, Z3A32, Z3A33, Z3A34, Z3A35, Z3A36, Z3A37, Z3A38, Z3A39, Z3A40, Z3A41, Z3A42, Z3A49
Primary immunodeficiencies/ Other IC	ICD-10-CM Dx	G113, D80, D800, D803, D804, D805, D806, D807, D81, D810, D811, D812, D815, D816, D817, D8189, D819, D82, D820, D821, D824, D83, D830, D831, D832, D838, D839, D8481, D822, D823, D828, D829, D84821, D849,
Respiratory TB	ICD-10-CM Dx	A150, A154, A155, A156, A157, A158, A159
Smoking, current and former	ICD-10-CM Dx	F17, F172, F1720, F17200, F17201, F17203, F17208, F17209, F1721, F17210, F17211, F17213, F17218, F17219, F1722, F17220, F17221, F17223, F17228, F17229, F1729, F17290, F17291, F17293, F17298, F17299, O9933, O99330, O99331, O99332, O99333, O99334, O99335, Z720, Z87891
Solid organ or blood stem cell transplantation	CPT	32851, 32852, 32853, 32854, 33935, 33945, 44135, 44136, 47135, 47136, 48554, 50360, 50365, 50370
	DRG	1, 2, 5, 6, 7, 8, 19, 650, 651, 652
	HCPCS	S2053, S2054, S2060, S2152, S2065
	ICD-10-CM Dx	Z4821, Z4822, Z4823, Z4824, Z48280, Z48288, Z940, Z941, Z942, Z943, Z944, Z9482, Z9483, T8610, T8619, T8620, T86298, T8630, T8639, T8640, T8649, T86818, T86819, T86858, T86859, T86898, T86899
	ICD-10-PCS	02YA0Z0, 02YA0Z1, 02YA0Z2, 07YM0Z0, 07YM0Z1, 07YM0Z2, 07YP0Z0, 07YP0Z1, 07YP0Z2, 0BYC0Z0, 0BYC0Z1, 0BYC0Z2, 0BYD0Z0, 0BYD0Z1, 0BYD0Z2, 0BYF0Z0, 0BYF0Z1, 0BYF0Z2, 0BYH0Z0, 0BYH0Z1, 0BYH0Z2, 0BYJ0Z0, 0BYJ0Z1, 0BYJ0Z2, 0BYK0Z0, 0BYK0Z1, 0BYK0Z2, 0BYL0Z0, 0BYL0Z1, 0BYL0Z2, 0BYM0Z0, 0BYM0Z1, 0BYM0Z2, 0DY50Z0, 0DY50Z1, 0DY50Z2, 0DY60Z0, 0DY60Z1, 0DY60Z2, 0DY80Z0, 0DY80Z1, 0DY80Z2, 0DYE0Z0, 0DYE0Z1, 0DYE0Z2, 0FY00Z0, 0FY00Z1, 0FY00Z2, 0FYG0Z0, 0FYG0Z1, 0FYG0Z2, 0TY00Z0, 0TY00Z1, 0TY00Z2, 0TY10Z0, 0TY10Z1, 0TY10Z2, BT2900Z, BT290ZZ, BT2910Z, BT291ZZ, BT29Y0Z, BT29YZZ, BT29ZZZ, BT39Y0Z, BT39YZZ, BT39ZZZ, BT49ZZZ
	CPT	38240, 38241, 38242, 38243

Stem cell transplantation	DRG	014, 016, 017
	HCPCS	S2142, S2150
	ICD-10-CM Dx	T8600, T8609, Z48290, Z9481
	ICD-10-PCS	30230AZ, 30230G1, 30230G2, 30230G3, 30230G4, 30230X1, 30230X2, 30230X3, 30230X4, 30230Y1, 30230Y2, 30230Y3, 30230Y4, 30233AZ, 30233G1, 30233G2, 30233G3, 30233G4, 30233X1, 30233X2, 30233X3, 30233X4, 30233Y1, 30233Y2, 30233Y3, 30233Y4, 30240AZ, 30240G1, 30240G2, 30240G3, 30240G4, 30240X1, 30240X2, 30240X3, 30240X4, 30240Y1, 30240Y2, 30240Y3, 30240Y4, 30243AZ, 30243G1, 30243G2, 30243G3, 30243G4, 30243X1, 30243X2, 30243X3, 30243X4, 30243Y1, 30243Y2, 30243Y3, 30243Y4, 30250G1, 30250X1, 30250Y1, 30253G1, 30253X1, 30253Y1, 30260G1, 30260X1, 30260Y1, 30263G1, 30263X1, 30263Y1
Use of immunosuppressive medications	NDC	abatacept, adalimumab, alefacept, alemtuzumab, anakinra, apremilast, azacitidine, azathioprine, baricitinib, basiliximab, belatacept, belimumab, bendamustine hcl, brodalumab, busulfan, canakinumab/pf, capecitabine, carmustine, certolizumab pegol, chlorambucil, cladribine, clofarabine, cyclophosphamide, cyclosporine, cyclosporine/chondroitin sulfate a sodium, cytarabine, dacarbazine, daclizumab, daunorubicin/cytarabine liposomal, decitabine, decitabine/cedazuridine, dimethyl fumarate, diroximel fumarate, eculizumab, efalizumab, emapalumab, etanercept, everolimus, fingolimod hcl, floxuridine, fludarabine phosphate, fluorouracil, gemcitabine hcl, golimumab, guselkumab, ifosfamide, ifosfamide/mesna, inebilizumab, infliximab, ixekizumab, leflunomide, lenalidomide, lomustine, melphalan, melphalan flufenamide hydrochloride, melphalan hcl/betadex sulfobutyl ether sodium, mercaptopurine, methotrexate, muromonab, mycophenolate mofetil, mycophenolate sodium, natalizumab, nelarabine, ocrelizumab, ofatumumab, ozanimod hydrochloride, pemetrexed disodium, pirfenidone, pomalidomide, pralatrexate, ravulizumab, rilonacept, risankizumab, sarilumab, satralizumab, secukinumab, siltuximab, siponimod, sirolimus, sirolimus protein, streptozocin, tacrolimus, tacrolimus/hyaluronate sodium/niacinamide, tacrolimus/niacinamide, temozolomide, temsirolimus, teprotumumab, teriflunomide, thalidomide, thiotepa, tildrakizumab, tocilizumab, tofacitinib citrate, upadacitinib, ustekinumab, vedolizumab, rituximab and hyaluronidase, rituximab, rituximab/hyaluronidase, human recombinant
	HCPCS	C9006, C9020, C9024, C9026, C9029, C9042, C9050, C9052, C9061, C9080, C9087, C9091, C9106, C9110, C9126, C9211, C9212, C9219, C9230, C9236, C9239, C9249, C9261, C9264, C9286, C9419, C9420, C9421, C9436, C9438, C9455, C9467, C9487, C9494, J0129, J0135, J0202, J0215, J0480, J0485, J0490, J0594, J0638, J0717, J0718, J0893, J0894, J1212, J1300, J1303, J1438, J1602, J1628, J1745, J1823, J2323, J2327, J2350, J2793, J2860, J3241, J3245, J3262, J3357, J3358, J3380, J7500, J7501, J7502, J7503, J7504, J7505, J7507, J7508, J7511, J7513, J7515, J7517, J7518, J7520, J7525, J7527, J8510, J8520, J8521, J8530, J8561, J8562, J8600, J8610, J8700, J9010, J9025, J9027, J9033, J9034, J9036, J9050, J9065, J9070, J9071, J9080, J9090, J9091, J9092, J9093, J9094, J9095, J9096, J9097, J9098, J9100, J9130, J9150, J9151, J9153, J9185, J9190, J9198, J9199, J9200, J9201, J9208, J9210, J9245, J9246, J9247, J9250, J9260, J9261, J9302, J9304, J9305, J9307, J9310, J9311, J9312, J9314, J9320, J9328, J9330, J9331, J9340, Q0249, Q2019, Q2044, Q4079, Q5102, Q5103, Q5104, Q5109, Q5115, Q5119, Q5121, Q5123, Q9979, Q9989, S0087, S0108, S0162, S0172, S0178, S0193, S9359

CPT, Current Procedural Terminology; HCPCS, Healthcare Common Procedure Coding System; ICD-9-CM, International Classification of Diseases, 9th Edition, Clinical Modification; ICD-10-CDM, International Classification of Diseases, 10th Edition, Clinical Modification; NDC, National Drug Codes; SNOMED, Systematized Nomenclature of Medicine

Supplementary Table S2. Baseline characteristics of the diabetic cohort. Data are presented as n (%) unless otherwise stated.

		Pre-weighting			Post-weighting		
		mRNA-1273.222	BNT162b2 Bivalent	SMD	mRNA-1273.222	BNT162b2 Bivalent	SMD
**Number of patients**		252,180	388,331		252,635	387,233	
**Age at index, mean (SD)**		66 (12.0)	66 (12.5)	0.0733	66 (12.3)	66 (12.3)	0.0022
**Sex**	Female	130,255 (51.7)	202,730 (52.2)	0.0111	131,339 (52.0)	201,368 (52.0)	0.0003
	Male	121,925 (48.3)	185,601 (47.8)		121,296 (48.0)	185,865 (48.0)	
**Race**	Black	22,407 (8.9)	37,608 (9.7)	0.0320	23,619 (9.3)	36,299 (9.4)	0.0009
	Other	18,681 (7.4)	28,055 (7.2)		18,498 (7.3)	28,302 (7.3)	
	White	114,654 (45.5)	172,396 (44.4)		113,246 (44.8)	173,458 (44.8)	
	Unknown	96,438 (38.2)	150,272 (38.7)		97,272 (38.5)	149,173 (38.5)	
**Ethnicity**	Hispanic	17,882 (7.1)	28,958 (7.5)	0.0190	18,395 (7.3)	28,308 (7.3)	0.0012
	Non-Hispanic	208,260 (82.6)	317,922 (81.9)		207,626 (82.2)	318,127 (82.2)	
	Unknown	26,037 (10.3)	41,450 (10.7)		26,614 (10.5)	40,798 (10.5)	
**Insurance Type**	Commercial	83,761 (33.2)	133,996 (34.5)	0.0620	86,711 (34.3)	132,187 (34.1)	0.0050
	Medicaid	31,879 (12.6)	55,033 (14.2)		34,418 (13.6)	52,783 (13.6)	
	Medicare Advantage	114,847 (45.5)	167,073 (43.0)		110,113 (43.6)	169,592 (43.8)	
	Medicare FFS	6,058 (2.4)	9,149 (2.4)		6,009 (2.4)	9,189 (2.4)	
	Other	14,927 (5.9)	22,084 (5.7)		14,715 (5.8)	22,453 (5.8)	
	Unknown	708 (0.3)	996 (0.3)		669 (0.3)	1,029 (0.3)	
**Region**	Midwest	44,220 (17.5)	84,251 (21.7)	0.1078	51,348 (20.3)	78,313 (20.2)	0.0027
	Northeast	67,466 (26.8)	102,595 (26.4)		66,843 (26.5)	102,704 (26.5)	
	South	80,866 (32.1)	115,840 (29.8)		77,173 (30.5)	118,457 (30.6)	
	West	48,673 (19.3)	70,084 (18.0)		46,771 (18.5)	71,673 (18.5)	
	Unknown	10,955 (4.3)	15,561 (4.0)		10,500 (4.2)	16,084 (4.2)	

		Pre-weighting			Post-weighting		
		mRNA-1273.222	BNT162b2 Bivalent	SMD	mRNA-1273.222	BNT162b2 Bivalent	SMD
**Month of index**	08-2022	3 (0.0)	4 (0.0)	0.0773	4 (0.0)	5 (0.0)	0.0019
	09-2022	51,298 (20.3)	89,983 (23.2)		55,586 (22.0)	85,418 (22.1)	
	10-2022	86,591 (34.3)	134,459 (34.6)		87,212 (34.5)	133,576 (34.5)	
	11-2022	54,239 (21.5)	77,752 (20.0)		52,089 (20.6)	79,758 (20.6)	
	12-2022	35,991 (14.3)	51,496 (13.3)		34,570 (13.7)	52,934 (13.7)	
	1-2023	17,302 (6.9)	24,580 (6.3)		16,529 (6.5)	25,346 (6.5)	
	2-2023	6,756 (2.7)	10,057 (2.6)		6,646 (2.6)	10,195 (2.6)	
**Place of service**	IP claim	1,092 (0.4)	2,589 (0.7)	0.0696	1,476 (0.6)	2,254 (0.6)	0.0014
	OP EHR	1,710 (0.7)	2,314 (0.6)		1,650 (0.7)	2,481 (0.6)	
	OP claim	31,400 (12.5)	56,314 (14.5)		34,746 (13.8)	53,329 (13.8)	
	Pharmacy claim	217,978 (86.4)	327,114 (84.2)		214,763 (85.0)	329,169 (85.0)	
**Number of OP visits**		2.0 (5.6)	2.0 (5.6)	0.0054	2.0 (5.6)	2.0 (5.6)	0.0010
**Number of hospitalizations**		0.3 (1.0)	0.3 (1.1)	0.0288	0.3 (1.1)	0.3 (1.1)	0.0010
**Primary series COVID-19 vaccine**	Heterologous	17,810 (7.1)	42,826 (11.0)	0.2290	24,537 (9.7)	37,072 (9.6)	0.0048
	Homologous	74,537 (29.6)	80,599 (20.8)		60,388 (23.9)	92,544 (23.9)	
	Not reported	159,833 (63.4)	264,906 (68.2)		167,710 (66.4)	257,616 (66.5)	
**Time since last monovalent COVID-19 vaccine**	≤90 days	4,421 (1.8)	5,241 (1.3)	0.2347	3,763 (1.5)	5,806 (1.5)	0.0019
	91–180 days	55,425 (22.0)	55,479 (14.3)		43,099 (17.1)	65,920 (17.0)	
	>180 days	140,328 (55.6)	220,215 (56.7)		142,425 (56.4)	218,640 (56.5)	
	Not reported	52,006 (20.6)	107,396 (27.7)		63,348 (25.1)	96,867 (25.0)	
**Time since last COVID-19 infection**	≤120 days	11,675 (4.6)	18,870 (4.9)	0.0419	12,110 (4.8)	18,532 (4.8)	0.0003
	121–180 days	5,728 (2.3)	8,729 (2.2)		5,739 (2.3)	8,773 (2.3)	
	>180 days	27,039 (10.7)	46,495 (12.0)		29,126 (11.5)	44,613 (11.5)	
	Not reported	207,738 (82.4)	314,237 (80.9)		205,661 (81.4)	315,314 (81.4)	

		Pre-weighting			Post-weighting		
		mRNA-1273.222	BNT162b2 Bivalent	SMD	mRNA-1273.222	BNT162b2 Bivalent	SMD
**Patients with underlying medical conditions**	Asthma	30,998 (12.3)	48,687 (12.5)	0.0074	31,517 (12.5)	48,262 (12.5)	0.0004
	Cancer	30,566 (12.1)	45,894 (11.8)	0.0093	30,037 (11.9)	46,129 (11.9)	0.0007
	Cerebrovascular disease	29,082 (11.5)	44,482 (11.5)	0.0024	28,868 (11.4)	44,397 (11.5)	0.0012
	Chronic kidney disease	55,668 (22.1)	86,549 (22.3)	0.0051	55,717 (22.1)	85,770 (22.1)	0.0023
	Chronic lung disease[a]	39,618 (15.7)	60,793 (15.7)	0.0015	39,481 (15.6)	60,650 (15.7)	0.0010
	Chronic liver disease	6,763 (2.7)	10,853 (2.8)	0.0069	6,947 (2.7)	10,662 (2.8)	0.0002
	Cystic fibrosis	107 (0.0)	136 (0.0)	0.0038	93 (0.0)	144 (0.0)	0.0002
	Diabetes type 1 or 2	252,180 (100)	388,331 (100)	0.0000	252,635 (100.0)	387,233 (100.0)	0.0000
	Disability	16,119 (6.4)	25,945 (6.7)	0.0117	16,661 (6.6)	25,488 (6.6)	0.0005
	Heart conditions	74,729 (29.6)	114,201 (29.4)	0.0049	74,237 (29.4)	114,055 (29.5)	0.0015
	HIV	1,890 (0.7)	3,012 (0.8)	0.0030	1,943 (0.8)	2,972 (0.8)	0.0002
	Mental health disorders	54,361 (21.6)	88,974 (22.9)	0.0326	56,693 (22.4)	86,851 (22.4)	0.0003
	Neurological conditions	11,470 (4.5)	19,591 (5.0)	0.0232	12,239 (4.8)	18,812 (4.9)	0.0006
	Obesity	99,267 (39.4)	156,515 (40.3)	0.0192	100,980 (40.0)	154,749 (40.0)	0.0002
	Primary immunodeficiencies	6,312 (2.5)	9,929 (2.6)	0.0034	6,311 (2.5)	9,758 (2.5)	0.0014
	Pregnancy[b]	194 (0.1)	369 (0.1)	0.0062	225 (0.1)	344 (0.1)	0.0001
	Physical inactivity	450 (0.2)	715 (0.2)	0.0013	463 (0.2)	707 (0.2)	0.0001
	Smoking[c]	50,802 (20.1)	80,654 (20.8)	0.0155	51,870 (20.5)	79,560 (20.5)	0.0004
	Solid organ or hematopoietic stem cell transplant	2,506 (1.0)	4,324 (1.1)	0.0117	2,675 (1.1)	4,129 (1.1)	0.0007
	Tuberculosis	179 (0.1)	288 (0.1)	0.0012	184 (0.1)	282 (0.1)	0.0001
	Use of immunosuppressants	16,511 (6.5)	24,735 (6.4)	0.0072	16,245 (6.4)	24,903 (6.4)	0.0000

IQR, interquartile range; SD, standard deviation; SMD, standardized mean difference

[a]except for asthma; [b]Includes recent pregnancy ; [c]Includes current and former smoker

Supplementary Table S3. Baseline characteristics the cerebro- and cardiovascular cohort. Data are presented as n (%) unless otherwise stated

		Pre-weighting			Post-weighting		
		**mRNA-1273.222**	**BNT162b2 Bivalent**	**SMD**	**mRNA-1273.222**	**BNT162b2 Bivalent**	**SMD**
**Number of patients**		204,933	311,148		205,364	310,143	
**Age at index, mean (SD)**		71 (10.9)	70 (11.5)	0.0398	70 (11.3)	70 (11.3)	0.0044
**Sex**	Female	95,401 (46.6)	147,760 (47.5)	0.0188	96,779 (47.1)	146,187 (47.1)	0.0002
	Male	109,532 (53.4)	163,388 (52.5)		108,585 (52.9)	163,956 (52.9)	
**Race**	Black	13,912 (6.8)	23,334 (7.5)	0.0320	14,801 (7.2)	22,413 (7.2)	0.0012
	Other	10,276 (5.0)	14,855 (4.8)		10,057 (4.9)	15,134 (4.9)	
	White	108,973 (53.2)	162,703 (52.3)		108,104 (52.6)	163,209 (52.6)	
	Unknown	71,772 (35.0)	110,256 (35.4)		72,402 (35.3)	109,388 (35.3)	
**Ethnicity**	Hispanic	9,457 (4.6)	15,246 (4.9)	0.0200	9,768 (4.8)	14,830 (4.8)	0.0010
	Non-Hispanic	173,674 (84.7)	261,476 (84.0)		173,228 (84.4)	261,527 (84.3)	
	Unknown	21,801 (10.6)	34,424 (11.1)		22,369 (10.9)	33,786 (10.9)	
**Insurance Type**	Commercial	54,509 (26.6)	84,041 (27.0)	0.0555	55,943 (27.2)	83,756 (27.0)	0.0062
	Medicaid	19,405 (9.5)	34,251 (11.0)		21,481 (10.5)	32,441 (10.5)	
	Medicare Advantage	114,368 (55.8)	168,737 (54.2)		111,619 (54.4)	169,397 (54.6)	
	Medicare FFS	5,684 (2.8)	8,567 (2.8)		5,680 (2.8)	8,548 (2.8)	
	Other	10,403 (5.1)	14,797 (4.8)		10,113 (4.9)	15,206 (4.9)	
	Unknown	564 (0.3)	755 (0.2)		529 (0.3)	794 (0.3)	
**Region**	Midwest	36,725 (17.9)	69,030 (22.2)	0.1126	42,760 (20.8)	64,179 (20.7)	0.0034
	Northeast	59,016 (28.8)	89,502 (28.8)		58,837 (28.7)	89,138 (28.7)	
	South	65,217 (31.8)	90,797 (29.2)		61,669 (30.0)	93,284 (30.1)	
	West	35,985 (17.6)	50,378 (16.2)		34,342 (16.7)	51,827 (16.7)	
	Unknown	7,990 (3.9)	11,441 (3.7)		7,756 (3.8)	11,715 (3.8)	

		Pre-weighting			Post-weighting		
		mRNA-1273.222	BNT162b2 Bivalent	SMD	mRNA-1273.222	BNT162b2 Bivalent	SMD
**Month of index**	08-2022	1 (0.0)	3 (0.0)	0.0814	2 (0.0)	3 (0.0)	0.0024
	09-2022	44,929 (21.9)	78,007 (25.1)		48,714 (23.7)	73,860 (23.8)	
	10-2022	73,195 (35.7)	111,005 (35.7)		73,308 (35.7)	110,622 (35.7)	
	11-2022	42,799 (20.9)	60,547 (19.5)		41,168 (20.0)	62,071 (20.0)	
	12-2022	26,942 (13.1)	37,760 (12.1)		25,850 (12.6)	38,949 (12.6)	
	1-2023	12,340 (6.0)	17,173 (5.5)		11,772 (5.7)	17,773 (5.7)	
	2-2023	4,727 (2.3)	6,653 (2.1)		4,550 (2.2)	6,865 (2.2)	
**Place of service**	IP claim	1,298 (0.6)	3,281 (1.1)	0.0854	1,835 (0.9)	2,785 (0.9)	0.0027
	OP EHR	1,516 (0.7)	2,134 (0.7)		1,510 (0.7)	2,237 (0.7)	
	OP claim	22,958 (11.2)	42,035 (13.5)		26,168 (12.7)	39,405 (12.7)	
	Pharmacy claim	179,161 (87.4)	263,698 (84.8)		175,851 (85.6)	265,716 (85.7)	
**Number of OP visit, mean (SD**		2.2 (5.9)	2.1 (6.0)	0.0059	2.2 (6.0)	2.2 (5.9)	0.0012
**Number of hospitalizations, mean (SD**		0.4 (1.1)	0.5 (1.3)	0.0374	0.4 (1.2)	0.4 (1.2)	0.0018
**Primary series COVID-19 vaccine**	Heterologous	12,811 (6.3)	31,402 (10.1)	0.2514	18,125 (8.8)	26,891 (8.7)	0.0060
	Homologous	58,425 (28.5)	58,712 (18.9)		45,951 (22.4)	69,271 (22.3)	
	Not reported	133,697 (65.2)	221,034 (71.0)		141,288 (68.8)	213,981 (69.0)	
**Time since last monovalent COVID-19 vaccine**	≤90 days	3,820 (1.9)	4,631 (1.5)	0.2451	3,324 (1.6)	5,045 (1.6)	0.0017
	91–180 days	49,819 (24.3)	50,960 (16.4)		39,502 (19.2)	59,555 (19.2)	
	>180 days	107,615 (52.5)	163,168 (52.4)		107,996 (52.6)	163,312 (52.7)	
	Not reported	43,679 (21.3)	92,389 (29.7)		54,542 (26.6)	82,231 (26.5)	
**Time since last COVID-19 infection**	≤120 days	10,697 (5.2)	17,139 (5.5)	0.0520	11,169 (5.4)	16,803 (5.4)	0.0012
	121–180 days	4,953 (2.4)	7,671 (2.5)		5,071 (2.5)	7,626 (2.5)	
	>180 days	20,862 (10.2)	36,399 (11.7)		22,909 (11.2)	34,568 (11.1)	
	Not reported	168,421 (82.2)	249,939 (80.3)		166,215 (80.9)	251,145 (81.0)	

		Pre-weighting			Post-weighting		
		mRNA-1273.222	BNT162b2 Bivalent	SMD	mRNA-1273.222	BNT162b2 Bivalent	SMD
**Patients with underlying medical conditions**	Asthma	26,063 (12.7)	39,893 (12.8)	0.0031	26,333 (12.8)	39,713 (12.8)	0.0005
	Cancer	32,176 (15.7)	48,150 (15.5)	0.0062	31,869 (15.5)	48,201 (15.5)	0.0006
	Cerebrovascular disease	68,237 (33.3)	104,581 (33.6)	0.0067	68,701 (33.5)	103,824 (33.5)	0.0005
	Chronic kidney disease	50,727 (24.8)	79,484 (25.6)	0.0183	51,500 (25.1)	78,103 (25.2)	0.0024
	Chronic lung disease[a]	51,577 (25.2)	79,663 (25.6)	0.0100	52,120 (25.4)	78,845 (25.4)	0.0010
	Chronic liver disease	5,045 (2.5)	8,046 (2.6)	0.0079	5,214 (2.5)	7,886 (2.5)	0.0002
	Cystic fibrosis	53 (0.0)	77 (0.0)	0.0007	52 (0.0)	78 (0.0)	0.0001
	Diabetes type 1 or 2	86,983 (42.4)	132,646 (42.6)	0.0038	87,283 (42.5)	131,963 (42.5)	0.0010
	Disability	16,094 (7.9)	25,275 (8.1)	0.0100	16,578 (8.1)	24,939 (8.0)	0.0011
	Heart conditions	169,131 (82.5)	256,250 (82.4)	0.0046	169,207 (82.4)	255,591 (82.4)	0.0004
	HIV	1,558 (0.8)	2,388 (0.8)	0.0008	1,581 (0.8)	2,380 (0.8)	0.0003
	Mental health disorders	45,569 (22.2)	75,331 (24.2)	0.0468	48,260 (23.5)	72,853 (23.5)	0.0002
	Neurological conditions	14,800 (7.2)	26,393 (8.5)	0.0469	16,361 (8.0)	24,804 (8.0)	0.0011
	Obesity	67,251 (32.8)	103,966 (33.4)	0.0127	68,200 (33.2)	102,982 (33.2)	0.0001
	Primary immunodeficiencies	5,888 (2.9)	9,138 (2.9)	0.0038	5,924 (2.9)	8,997 (2.9)	0.0010
	Pregnancy[b]	65 (0.0)	105 (0.0)	0.0011	68 (0.0)	102 (0.0)	0.0002
	Physical inactivity	398 (0.2)	655 (0.2)	0.0036	418 (0.2)	634 (0.2)	0.0002
	Smoking[c]	57,046 (27.8)	88,591 (28.5)	0.0141	58,015 (28.2)	87,619 (28.3)	<0.0001
	Solid organ or hematopoietic stem cell transplant	2,121 (1.0)	3,566 (1.1)	0.0107	2,258 (1.1)	3,424 (1.1)	0.0005
	Tuberculosis	151 (0.1)	250 (0.1)	0.0024	155 (0.1)	239 (0.1)	0.0006
	Use of immunosuppressants	15,135 (7.4)	22,498 (7.2)	0.0059	14,987 (7.3)	22,617 (7.3)	0.0002

IQR, interquartile range; SD, standard deviation; SMD, standardized mean difference

[a]except for asthma; [b]Includes recent pregnancy ; [c]Includes current and former smoker

Supplementary Table S4. Baseline characteristics of the chronic lung disease cohort. Data are presented as n (%) unless otherwise stated

		Pre-weighting			Post-weighting		
		**mRNA-1273.222**	**BNT162b2 Bivalent**	**SMD**	**mRNA-1273.222**	**BNT162b2 Bivalent**	**SMD**
**Number of patients**		183,686	290,887		183,969	290,178	
**Age at index, mean (SD)**		64 (15.1)	62 (16.1)	0.0912	63 (15.8)	63 (15.8)	0.0026
**Sex**	Female	113,666 (61.9)	182,671 (62.8)	0.0189	114,918 (62.5)	181,255 (62.5)	0.0001
	Male	70,020 (38.1)	108,216 (37.2)		69,051 (37.5)	108,923 (37.5)	
**Race**	Black	13,029 (7.1)	22,950 (7.9)	0.0364	13,904 (7.6)	22,012 (7.6)	0.0012
	Other	8,301 (4.5)	12,494 (4.3)		8,093 (4.4)	12,725 (4.4)	
	White	95,860 (52.2)	148,435 (51.0)		94,665 (51.5)	149,288 (51.4)	
	Unknown	66,496 (36.2)	107,008 (36.8)		67,307 (36.6)	106,154 (36.6)	
**Ethnicity**	Hispanic	9,245 (5.0)	15,312 (5.3)	0.0167	9,481 (5.2)	15,006 (5.2)	0.0009
	Non-Hispanic	154,732 (84.2)	243,269 (83.6)		154,340 (83.9)	243,361 (83.9)	
	Unknown	19,708 (10.7)	32,305 (11.1)		20,147 (11.0)	31,811 (11.0)	
**Insurance Type**	Commercial	65,102 (35.4)	106,048 (36.5)	0.0656	67,092 (36.5)	105,143 (36.2)	0.0059
	Medicaid	25,861 (14.1)	46,180 (15.9)		27,926 (15.2)	44,165 (15.2)	
	Medicare Advantage	77,978 (42.5)	115,539 (39.7)		74,198 (40.3)	117,673 (40.6)	
	Medicare FFS	4,723 (2.6)	7,231 (2.5)		4,671 (2.5)	7,323 (2.5)	
	Other	9,569 (5.2)	15,168 (5.2)		9,623 (5.2)	15,154 (5.2)	
	Unknown	453 (0.2)	721 (0.2)		457 (0.2)	719 (0.2)	
**Region**	Midwest	36,637 (19.9)	68,684 (23.6)	0.0932	41,223 (22.4)	64,820 (22.3)	0.0020
	Northeast	48,351 (26.3)	76,417 (26.3)		48,292 (26.3)	76,251 (26.3)	
	South	54,946 (29.9)	80,846 (27.8)		52,318 (28.4)	82,697 (28.5)	
	West	36,797 (20.0)	54,634 (18.8)		35,437 (19.3)	55,841 (19.2)	
	Unknown	6,955 (3.8)	10,306 (3.5)		6,699 (3.6)	10,569 (3.6)	

		Pre-weighting			Post-weighting		
		mRNA-1273.222	BNT162b2 Bivalent	SMD	mRNA-1273.222	BNT162b2 Bivalent	SMD
**Month of index**	08-2022	1 (0.0)	2 (0.0)	0.0735	1 (0.0)	2 (0.0)	0.0010
	09-2022	39,554 (21.5)	70,890 (24.4)		42,826 (23.3)	67,604 (23.3)	
	10-2022	63,747 (34.7)	100,777 (34.6)		63,794 (34.7)	100,576 (34.7)	
	11-2022	39,194 (21.3)	58,110 (20.0)		37,694 (20.5)	59,429 (20.5)	
	12-2022	24,553 (13.4)	36,429 (12.5)		23,662 (12.9)	37,300 (12.9)	
	1-2023	11,925 (6.5)	17,416 (6.0)		11,360 (6.2)	17,938 (6.2)	
	2-2023	4,712 (2.6)	7,263 (2.5)		4,632 (2.5)	7,330 (2.5)	
**Place of service**	IP claim	901 (0.5)	2,167 (0.7)	0.0882	1,202 (0.7)	1,893 (0.7)	0.0015
	OP EHR	1,343 (0.7)	1,916 (0.7)		1,298 (0.7)	2,020 (0.7)	
	OP claim	21,975 (12.0)	42,775 (14.7)		25,170 (13.7)	39,774 (13.7)	
	Pharmacy claim	159,467 (86.8)	244,029 (83.9)		156,300 (85.0)	246,491 (84.9)	
**Number of OP visits**		2.2 (6.2)	2.1 (6.1)	0.0108	2.2 (6.2)	2.2 (6.1)	0.0004
**Number of hospitalizations**		0.4 (1.1)	0.4 (1.3)	0.0251	0.4 (1.2)	0.4 (1.2)	0.0015
**Primary series COVID-19 vaccine**	Heterologous	15,291 (8.3)	33,332 (11.5)	0.1944	19,449 (10.6)	30,089 (10.4)	0.0065
	Homologous	55,141 (30.0)	64,353 (22.1)		45,629 (24.8)	72,181 (24.9)	
	Not reported	113,254 (61.7)	193,202 (66.4)		118,891 (64.6)	187,908 (64.8)	
**Time since last monovalent COVID-19 vaccine**	≤90 days	3,370 (1.8)	4,188 (1.4)	0.2265	2,894 (1.6)	4,591 (1.6)	0.0014
	91–180 days	39,362 (21.4)	40,922 (14.1)		30,724 (16.7)	48,378 (16.7)	
	>180 days	105,634 (57.5)	170,832 (58.7)		107,343 (58.3)	169,473 (58.4)	
	Not reported	35,320 (19.2)	74,945 (25.8)		43,007 (23.4)	67,736 (23.3)	
**Time since last COVID-19 infection**	≤120 days	10,893 (5.9)	17,979 (6.2)	0.0387	11,227 (6.1)	17,701 (6.1)	0.0003
	121–180 days	5,305 (2.9)	8,485 (2.9)		5,361 (2.9)	8,452 (2.9)	
	>180 days	21,288 (11.6)	37,109 (12.8)		22,727 (12.4)	35,813 (12.3)	
	Not reported	146,200 (79.6)	227,314 (78.1)		144,653 (78.6)	228,213 (78.6)	

		Pre-weighting			Post-weighting		
		**mRNA-1273.222**	**BNT162b2 Bivalent**	**SMD**	**mRNA-1273.222**	**BNT162b2 Bivalent**	**SMD**
**Patients with underlying medical conditions**	Asthma	107,684 (58.6)	175,269 (60.3)	0.0332	110,148 (59.9)	173,369 (59.7)	0.0026
	Cancer	24,604 (13.4)	37,661 (12.9)	0.0132	24,036 (13.1)	38,004 (13.1)	0.0009
	Cerebrovascular disease	21,797 (11.9)	33,620 (11.6)	0.0096	21,355 (11.6)	33,799 (11.6)	0.0012
	Chronic kidney disease	32,179 (17.8)	51,503 (17.7)	0.0028	32,362 (17.6)	51,318 (17.7)	0.0025
	Chronic lung disease[a]	98,839 (53.8)	150,479 (51.7)	0.0416	96,080 (52.2)	152,012 (52.4)	0.0032
	Chronic liver disease	4,553 (2.5)	7,519 (2.6)	0.0068	4,657 (2.5)	7,375 (2.5)	0.0006
	Cystic fibrosis	159 (0.1)	245 (0.1)	0.0008	156 (0.1)	245 (0.1)	<0.0001
	Diabetes type 1 or 2	60,381 (32.9)	93,809 (32.2)	0.0133	59,528 (32.4)	94,137 (32.4)	0.0018
	Disability	17,623 (9.6)	29,744 (10.2)	0.0211	18,393 (10.0)	29,018 (10.0)	0.0001
	Heart conditions	57,671 (31.4)	89,015 (30.6)	0.0172	56,501 (30.7)	89,461 (30.8)	0.0025
	HIV	1,607 (0.9)	2,513 (0.9)	0.0012	1,606 (0.9)	2,527 (0.9)	0.0002
	Mental health disorders	51,992 (28.3)	87,178 (30.0)	0.0366	53,982 (29.3)	85,193 (29.4)	0.0004
	Neurological conditions	8,827 (4.8)	15,499 (5.3)	0.0238	9,396 (5.1)	14,884 (5.1)	0.0010
	Obesity	65,971 (35.9)	105,405 (36.2)	0.0067	66,354 (36.1)	104,774 (36.1)	0.0008
	Primary immunodeficiencies	6,664 (3.6)	10,231 (3.5)	0.0060	6,473 (3.5)	10,272 (3.5)	0.0011
	Pregnancy[b]	477 (0.3)	935 (0.3)	0.0115	554 (0.3)	870 (0.3)	0.0002
	Physical inactivity	384 (0.2)	610 (0.2)	0.0001	383 (0.2)	607 (0.2)	0.0002
	Smoking[c]	56,782 (30.9)	89,609 (30.8)	0.0023	56,543 (30.7)	89,411 (30.8)	0.0017
	Solid organ or hematopoietic stem cell transplant	1,446 (0.8)	2,504 (0.9)	0.0081	1,516 (0.8)	2,414 (0.8)	0.0009
	Tuberculosis	182 (0.1)	289 (0.1)	0.0001	182 (0.1)	287 (0.1)	<0.0001
	Use of immunosuppressants	14,469 (7.9)	22,213 (7.6)	0.0090	14,193 (7.7)	22,400 (7.7)	0.0002

IQR, interquartile range; SD, standard deviation; SMD, standardized mean difference

[a]except for asthma; [b]Includes recent pregnancy ; [c]Includes current and former smoker

Supplementary Table S5. Baseline characteristics of the immunocompromised cohort. Data are presented as n (%) unless otherwise stated

		Pre-weighting			Post-weighting		
		mRNA-1273.222	BNT162b2 Bivalent	SMD	mRNA-1273.222	BNT162b2 Bivalent	SMD
**Number of patients**		169,185	258,467		169,509	257,751	
**Age at index, mean (SD)**		66 (13.1)	65 (13.6)	0.0763	65 (13.4)	65 (13.4)	0.0028
**Sex**	Female	93,938 (55.5)	145,949 (56.5)	0.0190	95,086 (56.1)	144,623 (56.1)	0.0003
	Male	75,247 (44.5)	112,518 (43.5)		74,423 (43.9)	113,128 (43.9)	
**Race**	Black	10,464 (6.2)	18,188 (7.0)	0.0408	11,311 (6.7)	17,284 (6.7)	0.0017
	Other	8,572 (5.1)	12,286 (4.8)		8,291 (4.9)	12,585 (4.9)	
	White	89,479 (52.9)	133,780 (51.8)		88,531 (52.2)	134,517 (52.2)	
	Unknown	60,670 (35.9)	94,213 (36.5)		61,376 (36.2)	93,365 (36.2)	
**Ethnicity**	Hispanic	7,823 (4.6)	12,027 (4.7)	0.0159	7,824 (4.6)	11,939 (4.6)	0.0006
	Non-Hispanic	142,195 (84.0)	215,861 (83.5)		141,982 (83.8)	215,836 (83.7)	
	Unknown	19,167 (11.3)	30,576 (11.8)		19,704 (11.6)	29,976 (11.6)	
**Insurance Type**	Commercial	64,096 (37.9)	101,719 (39.4)	0.0576	66,475 (39.2)	100,451 (39.0)	0.0058
	Medicaid	13,640 (8.1)	23,449 (9.1)		14,719 (8.7)	22,431 (8.7)	
	Medicare Advantage	76,348 (45.1)	110,638 (42.8)		73,265 (43.2)	112,072 (43.5)	
	Medicare FFS	3,410 (2.0)	4,570 (1.8)		3,156 (1.9)	4,782 (1.9)	
	Other	11,262 (6.7)	17,468 (6.8)		11,477 (6.8)	17,382 (6.7)	
	Unknown	429 (0.3)	623 (0.2)		417 (0.2)	633 (0.2)	
**Region**	Midwest	30,527 (18.0)	56,105 (21.7)	0.0951	34,769 (20.5)	52,634 (20.4)	0.0025
	Northeast	45,788 (27.1)	69,310 (26.8)		45,502 (26.8)	69,309 (26.9)	
	South	51,420 (30.4)	73,884 (28.6)		49,379 (29.1)	75,237 (29.2)	
	West	35,446 (21.0)	50,447 (19.5)		34,010 (20.1)	51,681 (20.1)	
	Unknown	6,004 (3.5)	8,721 (3.4)		5,849 (3.5)	8,890 (3.4)	

		Pre-weighting			Post-weighting		
		mRNA-1273.222	BNT162b2 Bivalent	SMD	mRNA-1273.222	BNT162b2 Bivalent	SMD
**Month of index**	08-2022	1 (0.0)	3 (0.0)	0.0689	2 (0.0)	3 (0.0)	0.0010
	09-2022	39,363 (23.3)	66,977 (25.9)		42,141 (24.9)	64,151 (24.9)	
	10-2022	59,500 (35.2)	91,220 (35.3)		59,780 (35.3)	90,820 (35.2)	
	11-2022	35,176 (20.8)	50,397 (19.5)		33,942 (20.0)	51,557 (20.0)	
	12-2022	21,822 (12.9)	30,902 (12.0)		20,875 (12.3)	31,757 (12.3)	
	1-2023	9,618 (5.7)	13,644 (5.3)		9,198 (5.4)	14,014 (5.4)	
	2-2023	3,705 (2.2)	5,324 (2.1)		3,572 (2.1)	5,449 (2.1)	
**Place of service**	IP claim	413 (0.2)	951 (0.4)	0.0683	539 (0.3)	829 (0.3)	0.0012
	OP EHR	1,090 (0.6)	1,610 (0.6)		1,095 (0.6)	1,649 (0.6)	
	OP claim	17,354 (10.3)	31,702 (12.3)		19,561 (11.5)	29,749 (11.5)	
	Pharmacy claim	150,328 (88.9)	224,204 (86.7)		148,313 (87.5)	225,524 (87.5)	
**Number of OP visits**		2.1 (5.7)	2.0 (5.6)	0.0115	2.0 (5.7)	2.0 (5.6)	0.0008
**Number of hospitalizations**		0.3 (0.9)	0.3 (1.0)	0.0234	0.3 (1.0)	0.3 (1.0)	0.0012
**Primary series COVID-19 vaccine**	Heterologous	12,436 (7.4)	26,239 (10.2)	0.2028	15,885 (9.4)	23,649 (9.2)	0.0066
	Homologous	49,937 (29.5)	54,850 (21.2)		40,900 (24.1)	62,249 (24.2)	
	Not reported	106,812 (63.1)	177,378 (68.6)		112,724 (66.5)	171,853 (66.7)	
**Time since last monovalent COVID-19 vaccine**	≤90 days	3,079 (1.8)	3,676 (1.4)	0.2498	2,653 (1.6)	4,054 (1.6)	0.0011
	91–180 days	41,132 (24.3)	41,064 (15.9)		32,195 (19.0)	48,836 (18.9)	
	>180 days	93,746 (55.4)	146,846 (56.8)		95,584 (56.4)	145,447 (56.4)	
	Not reported	31,228 (18.5)	66,881 (25.9)		39,077 (23.1)	59,414 (23.1)	
**Time since last COVID-19 infection**	≤120 days	7,844 (4.6)	12,398 (4.8)	0.0300	8,062 (4.8)	12,248 (4.8)	0.0005
	121–180 days	4,075 (2.4)	6,302 (2.4)		4,127 (2.4)	6,270 (2.4)	
	>180 days	15,539 (9.2)	25,877 (10.0)		16,483 (9.7)	25,045 (9.7)	
	Not reported	141,727 (83.8)	213,890 (82.8)		140,838 (83.1)	214,188 (83.1)	

		Pre-weighting			Post-weighting		
		mRNA-1273.222	BNT162b2 Bivalent	SMD	mRNA-1273.222	BNT162b2 Bivalent	SMD
**Patients with underlying medical conditions**	Asthma	20,039 (11.8)	31,209 (12.1)	0.0071	20,347 (12.0)	30,931 (12.0)	0.0001
	Cancer	99,704 (58.9)	151,325 (58.5)	0.0078	99,361 (58.6)	151,212 (58.7)	0.0010
	Cerebrovascular disease	16,402 (9.7)	24,583 (9.5)	0.0062	16,127 (9.5)	24,622 (9.6)	0.0013
	Chronic kidney disease	29,405 (17.4)	45,001 (17.4)	0.0008	29,223 (17.2)	44,691 (17.3)	0.0026
	Chronic lung disease[a]	27,936 (16.5)	41,819 (16.2)	0.0090	27,489 (16.2)	41,951 (16.3)	0.0016
	Chronic liver disease	4,246 (2.5)	6,803 (2.6)	0.0077	4,350 (2.6)	6,652 (2.6)	0.0009
	Cystic fibrosis	95 (0.1)	131 (0.1)	0.0024	88 (0.1)	135 (0.1)	0.0001
	Diabetes type 1 or 2	49,366 (29.2)	74,223 (28.7)	0.0102	48,735 (28.8)	74,326 (28.8)	0.0019
	Disability	12,658 (7.5)	19,945 (7.7)	0.0089	12,944 (7.6)	19,668 (7.6)	0.0002
	Heart conditions	40,636 (24.0)	60,807 (23.5)	0.0116	39,987 (23.6)	60,993 (23.7)	0.0017
	HIV	7,327 (4.3)	11,394 (4.4)	0.0038	7,467 (4.4)	11,324 (4.4)	0.0006
	Mental health disorders	33,435 (19.8)	54,152 (21.0)	0.0295	34,736 (20.5)	52,860 (20.5)	0.0004
	Neurological conditions	6,003 (3.5)	9,801 (3.8)	0.0130	6,221 (3.7)	9,521 (3.7)	0.0013
	Obesity	44,183 (26.1)	68,847 (26.6)	0.0118	44,742 (26.4)	68,124 (26.4)	0.0008
	Primary immunodeficiencies	15,989 (9.5)	25,055 (9.7)	0.0083	16,167 (9.5)	24,678 (9.6)	0.0013
	Pregnancy[b]	182 (0.1)	357 (0.1)	0.0087	215 (0.1)	327 (0.1)	<0.0001
	Physical inactivity	265 (0.2)	385 (0.1)	0.0020	259 (0.2)	392 (0.2)	0.0002
	Smoking[c]	34,740 (20.5)	54,411 (21.1)	0.0128	35,290 (20.8)	53,733 (20.8)	0.0007
	Solid organ or hematopoietic stem cell transplant	5,078 (3.0)	8,679 (3.4)	0.0203	5,435 (3.2)	8,307 (3.2)	0.0009
	Tuberculosis	160 (0.1)	228 (0.1)	0.0021	154 (0.1)	233 (0.1)	0.0001
	Use of immunosuppressants	63,796 (37.7)	98,130 (38.0)	0.0053	64,308 (37.9)	97,664 (37.9)	0.0010

IQR, interquartile range; SD, standard deviation; SMD, standardized mean difference

[a]except for asthma; [b]Includes recent pregnancy ; [c]Includes current and former smoker

Supplementary Table S6. Baseline characteristics of the chronic kidney disease cohort. Data are presented as n (%) unless otherwise stated

		Pre-weighting			Post-weighting		
		**mRNA-1273.222**	**BNT162b2 Bivalent**	**SMD**	**mRNA-1273.222**	**BNT162b2 Bivalent**	**SMD**
**Number of patients**		106,577	164,005		106,841	163,426	
**Age at index, mean (SD)**		72 (10.9)	72 (11.5)	0.0442	72 (11.3)	72 (11.3)	0.0042
**Sex**	Female	55,175 (51.8)	86,166 (52.5)	0.0154	55,801 (52.2)	85,380 (52.2)	0.0003
	Male	51,402 (48.2)	77,839 (47.5)		51,041 (47.8)	78,046 (47.8)	
**Race**	Black	10,048 (9.4)	17,304 (10.6)	0.0428	10,814 (10.1)	16,559 (10.1)	0.0010
	Other	6,171 (5.8)	8,781 (5.4)		5,900 (5.5)	9,019 (5.5)	
	White	53,503 (50.2)	80,686 (49.2)		53,018 (49.6)	81,030 (49.6)	
	Unknown	36,855 (34.6)	57,234 (34.9)		37,110 (34.7)	56,817 (34.8)	
**Ethnicity**	Hispanic	6,279 (5.9)	10,291 (6.3)	0.0184	6,486 (6.1)	9,994 (6.1)	0.0021
	Non-Hispanic	89,056 (83.6)	136,023 (82.9)		88,935 (83.2)	135,963 (83.2)	
	Unknown	11,241 (10.5)	17,690 (10.8)		11,421 (10.7)	17,469 (10.7)	
**Insurance Type**	Commercial	22,164 (20.8)	34,627 (21.1)	0.0534	22,777 (21.3)	34,501 (21.1)	0.0066
	Medicaid	9,583 (9.0)	17,186 (10.5)		10,698 (10.0)	16,297 (10.0)	
	Medicare Advantage	66,526 (62.4)	99,905 (60.9)		65,156 (61.0)	100,144 (61.3)	
	Medicare FFS	3,031 (2.8)	4,604 (2.8)		3,020 (2.8)	4,605 (2.8)	
	Other	4,999 (4.7)	7,290 (4.4)		4,928 (4.6)	7,477 (4.6)	
	Unknown	274 (0.3)	393 (0.2)		263 (0.2)	402 (0.2)	
**Region**	Midwest	18,149 (17.0)	35,666 (21.7)	0.1266	21,665 (20.3)	32,862 (20.1)	0.0045
	Northeast	26,045 (24.4)	40,073 (24.4)		26,002 (24.3)	39,905 (24.4)	
	South	34,184 (32.1)	49,399 (30.1)		32,720 (30.6)	50,212 (30.7)	
	West	23,678 (22.2)	32,228 (19.7)		22,028 (20.6)	33,672 (20.6)	
	Unknown	4,521 (4.2)	6,639 (4.0)		4,426 (4.1)	6,776 (4.1)	

		Pre-weighting			Post-weighting		
		mRNA-1273.222	BNT162b2 Bivalent	SMD	mRNA-1273.222	BNT162b2 Bivalent	SMD
**Month of index**	08-2022	2 (0.0)	0 (0.0)	0.0739	1 (0.0)	0 (0.0)	0.0020
	09-2022	22,466 (21.1)	39,055 (23.8)		24,214 (22.7)	37,146 (22.7)	
	10-2022	37,852 (35.5)	58,632 (35.8)		38,172 (35.7)	58,294 (35.7)	
	11-2022	22,819 (21.4)	32,453 (19.8)		21,762 (20.4)	33,308 (20.4)	
	12-2022	14,240 (13.4)	20,544 (12.5)		13,746 (12.9)	21,026 (12.9)	
	1-2023	6,556 (6.2)	9,446 (5.8)		6,352 (5.9)	9,699 (5.9)	
	2-2023	2,642 (2.5)	3,875 (2.4)		2,594 (2.4)	3,954 (2.4)	
**Place of service**	IP claim	697 (0.7)	1,796 (1.1)	0.1083	1,010 (0.9)	1,529 (0.9)	0.0034
	OP EHR	981 (0.9)	1,319 (0.8)		957 (0.9)	1,427 (0.9)	
	OP claim	12,669 (11.9)	24,798 (15.1)		14,891 (13.9)	22,780 (13.9)	
	Pharmacy claim	92,230 (86.5)	136,092 (83.0)		89,984 (84.2)	137,690 (84.3)	
**Number of OP visit, mean (SD**		2.4 (6.4)	2.4 (6.4)	0.0110	2.4 (6.5)	2.4 (6.4)	0.0018
**Number of hospitalizations, mean (SD**		0.4 (1.2)	0.5 (1.4)	0.0444	0.5 (1.3)	0.5 (1.3)	0.0016
**Primary series COVID-19 vaccine**	Heterologous	6,281 (5.9)	16,503 (10.1)	0.2721	9,266 (8.7)	13,923 (8.5)	0.0056
	Homologous	30,336 (28.5)	29,796 (18.2)		23,369 (21.9)	35,692 (21.8)	
	Not reported	69,960 (65.6)	117,706 (71.8)		74,206 (69.5)	113,811 (69.6)	
**Time since last monovalent COVID-19 vaccine**	≤90 days	2,027 (1.9)	2,405 (1.5)	0.2622	1,728 (1.6)	2,658 (1.6)	0.0024
	91–180 days	26,028 (24.4)	26,446 (16.1)		20,389 (19.1)	31,146 (19.1)	
	>180 days	55,143 (51.7)	83,906 (51.2)		54,996 (51.5)	84,273 (51.6)	
	Not reported	23,379 (21.9)	51,248 (31.2)		29,729 (27.8)	45,348 (27.7)	
**Time since last COVID-19 infection**	≤120 days	5,460 (5.1)	8,847 (5.4)	0.0571	5,701 (5.3)	8,684 (5.3)	0.0015
	121–180 days	2,442 (2.3)	3,769 (2.3)		2,459 (2.3)	3,760 (2.3)	
	>180 days	10,775 (10.1)	19,368 (11.8)		11,974 (11.2)	18,293 (11.2)	
	Not reported	87,900 (82.5)	132,021 (80.5)		86,707 (81.2)	132,688 (81.2)	

		Pre-weighting			Post-weighting		
		**mRNA-1273.222**	**BNT162b2 Bivalent**	**SMD**	**mRNA-1273.222**	**BNT162b2 Bivalent**	**SMD**
**Patients with underlying medical conditions**	Asthma	12,802 (12.0)	20,131 (12.3)	0.0080	13,052 (12.2)	19,930 (12.2)	0.0006
	Cancer	18,400 (17.3)	27,930 (17.0)	0.0062	18,224 (17.1)	27,934 (17.1)	0.0010
	Cerebrovascular disease	17,516 (16.4)	27,668 (16.9)	0.0117	17,757 (16.6)	27,258 (16.7)	0.0016
	Chronic kidney disease	106,577 (100)	164,005 (100)	0.0000	106,841 (100.0)	163,426 (100.0)	0.0000
	Chronic lung disease[a]	25,519 (23.9)	40,392 (24.6)	0.0160	25,996 (24.3)	39,811 (24.4)	0.0007
	Chronic liver disease	3,052 (2.9)	4,920 (3.0)	0.0081	3,165 (3.0)	4,836 (3.0)	0.0002
	Cystic fibrosis	44 (0.0)	67 (0.0)	0.0002	42 (0.0)	66 (0.0)	0.0005
	Diabetes type 1 or 2	55,668 (52.2)	86,549 (52.8)	0.0108	56,099 (52.5)	85,906 (52.6)	0.0012
	Disability	8,270 (7.8)	13,112 (8.0)	0.0087	8,492 (7.9)	12,949 (7.9)	0.0009
	Heart conditions	44,603 (41.9)	69,830 (42.6)	0.0147	45,085 (42.2)	69,084 (42.3)	0.0015
	HIV	1,207 (1.1)	1,839 (1.1)	0.0011	1,217 (1.1)	1,850 (1.1)	0.0007
	Mental health disorders	25,605 (24.0)	42,584 (26.0)	0.0448	27,025 (25.3)	41,286 (25.3)	0.0007
	Neurological conditions	8,552 (8.0)	14,990 (9.1)	0.0398	9,329 (8.7)	14,265 (8.7)	0.0001
	Obesity	38,388 (36.0)	60,064 (36.6)	0.0126	38,994 (36.5)	59,548 (36.4)	0.0012
	Primary immunodeficiencies	5,242 (4.9)	8,491 (5.2)	0.0118	5,358 (5.0)	8,266 (5.1)	0.0020
	Pregnancy[b]	20 (0.0)	45 (0.0)	0.0057	26 (0.0)	40 (0.0)	0.0001
	Physical inactivity	239 (0.2)	356 (0.2)	0.0015	234 (0.2)	360 (0.2)	0.0002
	Smoking[c]	25,610 (24.0)	40,754 (24.8)	0.0191	26,202 (24.5)	40,124 (24.6)	0.0006
	Solid organ or hematopoietic stem cell transplant	3,115 (2.9)	5,290 (3.2)	0.0175	3,324 (3.1)	5,100 (3.1)	0.0005
	Tuberculosis	68 (0.1)	144 (0.1)	0.0087	82 (0.1)	128 (0.1)	0.0005
	Use of immunosuppressants	9,460 (8.9)	14,522 (8.9)	0.0008	9,485 (8.9)	14,494 (8.9)	0.0003

IQR, interquartile range; SD, standard deviation; SMD, standardized mean difference

Supplementary Table S7: Unweighted, unadjusted relative vaccine effectiveness (rVE) estimates

	**COVID-19-related hospitalization**	**COVID-19-related outpatient encounter**
	rVE (95% CI)	rVE (95% CI)
Overall	12.7% (8.1%-17.0%)	2.9% (1.4%-4.4%)
Subgroup analyses		
Diabetes	19.8% (13.7%-25.4%)	5.3% (2.8%-7.7%)
Cerebro- and cardiovascular disease	21.0% (15.6%-26.1%)	10.6% (8.1%-12.9%)
Chronic lung disease	14.8% (8.2%-20.9%)	4.3% (1.6%-6.8%)
Immunocompromised	16.5% (8.8%-23.6%)	5.4% (2.4%-8.3%)
Chronic kidney disease	15.7% (8.4%-22.5%)	10.6% (7.2%-14.0%)
Sensitivity analyses		
Open claims	14.1% (11.3%-16.7%)	7.2% (6.3%-8.1%)
Closed claims – cut-off 28 Feb 23	14.6% (9.3%-19.6%)	2.6% (0.9%-4.3%)

Supplementary Table S8. Baseline characteristics of individuals included in the open claims sensitivity analysis. Data are presented as n (%) unless otherwise stated

		Pre-weighting			Post-weighting		
		**mRNA-1273.222**	**BNT162b2 Bivalent**	**SMD**	**mRNA-1273.222**	**BNT162b2 Bivalent**	**SMD**
**Number of patients**		1,960,185	2,969,597		1,964,137	2,961,234	
**Age at index, mean (SD)**		66 (14.9)	64 (16.0)	0.1097	65 (15.7)	65 (15.6)	0.0030
**Sex**	Female	1,101,985 (56.2)	1,700,044 (57.2)	0.0208	1,116,624 (56.9)	1,683,810 (56.9)	0.0002
	Male	858,200 (43.8)	1,269,553 (42.8)		847,513 (43.1)	1,277,425 (43.1)	
**Race**	Black	120,008 (6.1)	186,674 (6.3)	0.0286	121,625 (6.2)	184,059 (6.2)	0.0014
	Other	85,321 (4.4)	128,131 (4.3)		85,509 (4.4)	128,621 (4.3)	
	White	1,068,365 (54.5)	1,578,195 (53.1)		1,054,386 (53.7)	1,589,130 (53.7)	
	Unknown	686,491 (35.0)	1,076,597 (36.3)		702,616 (35.8)	1,059,424 (35.8)	
**Ethnicity**	Hispanic	77,991 (4.0)	128,833 (4.3)	0.0257	82,148 (4.2)	124,319 (4.2)	0.0011
	Non-Hispanic	1,649,332 (84.1)	2,471,516 (83.2)		1,642,149 (83.6)	2,475,085 (83.6)	
	Unknown	232,848 (11.9)	369,221 (12.4)		239,840 (12.2)	361,831 (12.2)	
**Insurance Type**	Commercial	552,012 (28.2)	897,051 (30.2)	0.0716	584,112 (29.7)	875,209 (29.6)	0.0044
	Medicaid	101,756 (5.2)	183,703 (6.2)		114,435 (5.8)	172,520 (5.8)	
	Medicare Advantage	350,121 (17.9)	512,954 (17.3)		340,184 (17.3)	516,148 (17.4)	
	Medicare FFS	16,044 (0.8)	24,479 (0.8)		16,126 (0.8)	24,296 (0.8)	
	Other	59,136 (3.0)	95,295 (3.2)		62,075 (3.2)	93,250 (3.1)	
	Unknown	881,116 (45.0)	1,256,115 (42.3)		847,205 (43.1)	1,279,811 (43.2)	
**Region**	Midwest	387,534 (19.8)	714,791 (24.1)	0.1365	445,123 (22.7)	667,829 (22.6)	0.0027
	Northeast	382,663 (19.5)	601,138 (20.2)		392,031 (20.0)	592,049 (20.0)	
	South	732,186 (37.4)	939,965 (31.7)		660,096 (33.6)	996,832 (33.7)	
	West	390,534 (19.9)	616,754 (20.8)		401,658 (20.4)	605,959 (20.5)	
	Unknown	67,268 (3.4)	96,949 (3.3)		65,228 (3.3)	98,567 (3.3)	

		Pre-weighting			Post-weighting		
		mRNA-1273.222	BNT162b2 Bivalent	SMD	mRNA-1273.222	BNT162b2 Bivalent	SMD
**Month of index**	08-2022	26 (0.0)	51 (0.0)	0.0961	33 (0.0)	47 (0.0)	0.0015
	09-2022	422,860 (21.6)	741,749 (25.0)		464,878 (23.7)	701,215 (23.7)	
	10-2022	686,911 (35.0)	1,060,697 (35.7)		695,856 (35.4)	1,049,508 (35.4)	
	11-2022	420,241 (21.4)	579,871 (19.5)		398,247 (20.3)	599,930 (20.3)	
	12-2022	261,175 (13.3)	356,695 (12.0)		246,321 (12.5)	370,893 (12.5)	
	1-2023	122,048 (6.2)	164,472 (5.5)		113,856 (5.8)	171,758 (5.8)	
	2-2023	46,924 (2.4)	66,062 (2.2)		44,946 (2.3)	67,883 (2.3)	
**Place of service**	IP claim	5,716 (0.3)	11,248 (0.4)	0.0702	6,834 (0.3)	10,281 (0.3)	0.0021
	OP EHR	47,934 (2.4)	63,041 (2.1)		45,604 (2.3)	67,788 (2.3)	
	OP claim	211,926 (10.8)	384,009 (12.9)		238,612 (12.1)	360,295 (12.2)	
	Pharmacy claim	1,694,609 (86.5)	2,511,299 (84.6)		1,673,087 (85.2)	2,522,872 (85.2)	
**Number of OP visits, mean (SD)**		2.4 (6.2)	2.3 (6.0)	0.0137	2.4 (6.2)	2.4 (6.1)	0.0012
**Number of hospitalizations, mean (SD)**		0.2 (0.7)	0.2 (0.8)	0.0104	0.2 (0.8)	0.2 (0.8)	0.0004
**Primary series COVID-19 vaccine**	Heterologous	128,691 (6.6)	302,676 (10.2)	0.2330	176,277 (9.0)	261,903 (8.8)	0.0046
	Homologous	563,137 (28.7)	585,351 (19.7)		450,126 (22.9)	679,068 (22.9)	
	Not reported	1,268,357 (64.7)	2,081,570 (70.1)		1,337,733 (68.1)	2,020,264 (68.2)	
**Time since last monovalent COVID-19 vaccine**	≤90 days	29,193 (1.5)	37,634 (1.3)	0.1523	26,369 (1.3)	39,918 (1.3)	0.0011
	91–180 days	377,459 (19.3)	430,909 (14.5)		317,273 (16.2)	478,643 (16.2)	
	>180 days	1,083,774 (55.3)	1,642,034 (55.3)		1,087,793 (55.4)	1,640,169 (55.4)	
	Not reported	469,759 (24.0)	859,020 (28.9)		532,703 (27.1)	802,504 (27.1)	
**Time since last COVID-19 infection**	≤120 days	70,375 (3.6)	111,823 (3.8)	0.0339	73,033 (3.7)	109,916 (3.7)	0.0009
	121–180 days	35,365 (1.8)	55,751 (1.9)		36,615 (1.9)	54,994 (1.9)	
	>180 days	143,640 (7.3)	242,213 (8.2)		154,766 (7.9)	232,819 (7.9)	
	Not reported	1,710,805 (87.3)	2,559,810 (86.2)		1,699,724 (86.5)	2,563,505 (86.6)	

		Pre-weighting			Post-weighting		
		mRNA-1273.222	BNT162b2 Bivalent	SMD	mRNA-1273.222	BNT162b2 Bivalent	SMD
**Patients with underlying medical conditions**	Asthma	239,547 (12.2)	378,092 (12.7)	0.0155	246,885 (12.6)	371,851 (12.6)	0.0004
	Cancer	274,094 (14.0)	397,098 (13.4)	0.0178	266,564 (13.6)	402,460 (13.6)	0.0006
	Cerebrovascular disease	182,716 (9.3)	263,756 (8.9)	0.0153	176,864 (9.0)	267,360 (9.0)	0.0008
	Chronic kidney disease	311,420 (15.9)	454,246 (15.3)	0.0163	302,912 (15.4)	458,357 (15.5)	0.0016
	Chronic lung disease[a]	279,050 (14.2)	396,105 (13.3)	0.0260	267,171 (13.6)	403,897 (13.6)	0.0011
	Chronic liver disease	34,511 (1.8)	52,909 (1.8)	0.0016	34,759 (1.8)	52,529 (1.8)	0.0003
	Cystic fibrosis	567 (0.0)	871 (0.0)	0.0002	575 (0.0)	867 (0.0)	<0.0001
	Diabetes type 1 or 2	672,173 (34.3)	978,019 (32.9)	0.0287	654,522 (33.3)	989,186 (33.4)	0.0017
	Disability	169,788 (8.7)	285,896 (9.6)	0.0335	183,394 (9.3)	275,196 (9.3)	0.0015
	Heart conditions	475,711 (24.3)	679,657 (22.9)	0.0325	457,340 (23.3)	691,483 (23.4)	0.0016
	HIV	14,635 (0.7)	21,945 (0.7)	0.0009	14,702 (0.7)	22,066 (0.7)	0.0004
	Mental health disorders	455,446 (23.2)	751,226 (25.3)	0.0481	483,565 (24.6)	727,306 (24.6)	0.0014
	Neurological conditions	80,821 (4.1)	133,357 (4.5)	0.0181	85,055 (4.3)	128,647 (4.3)	0.0007
	Obesity	573,660 (29.3)	882,196 (29.7)	0.0097	580,259 (29.5)	875,008 (29.5)	0.0001
	Primary immunodeficiencies	36,939 (1.9)	55,914 (1.9)	0.0001	36,724 (1.9)	55,583 (1.9)	0.0005
	Pregnancy[b]	9,095 (0.5)	17,848 (0.6)	0.0188	10,969 (0.6)	16,367 (0.6)	0.0008
	Physical inactivity	2,913 (0.1)	4,638 (0.2)	0.0019	3,071 (0.2)	4,586 (0.2)	0.0004
	Smoking[c]	373,645 (19.1)	566,861 (19.1)	0.0007	374,189 (19.1)	564,840 (19.1)	0.0006
	Solid organ or hematopoietic stem cell transplant	13,433 (0.7)	21,637 (0.7)	0.0052	13,953 (0.7)	21,098 (0.7)	0.0002
	Tuberculosis	945 (0.0)	1,504 (0.1)	0.0011	972 (0.0)	1,468 (0.0)	<0.0001
	Use of immunosuppressants	151,757 (7.7)	225,188 (7.6)	0.0060	149,962 (7.6)	226,206 (7.6)	0.0001

IQR, interquartile range; SD, standard deviation; SMD, standardized mean difference
[a]except for asthma; [b]Includes recent pregnancy ; [c]Includes current and former smoker

Supplementary Table S9. Baseline characteristics of individuals included in the sensitivity analysis using an end date of February 28, 2023. Data are presented as n (%) unless otherwise stated

		Pre-weighting			Post-weighting		
		mRNA-1273.222	BNT162b2 Bivalent	SMD	mRNA-1273.222	BNT162b2 Bivalent	SMD
**Number of patients**		748,343	1,190,324		749,557	1,187,593	
**Age at index, mean (SD)**		62 (15.6)	61 (16.4)	0.0954	61 (16.1)	61 (16.1)	0.0027
**Sex**	Female	425,872 (56.9)	689,028 (57.9)	0.0198	431,148 (57.5)	683,166 (57.5)	0.0001
	Male	322,471 (43.1)	501,296 (42.1)		318,409 (42.5)	504,427 (42.5)	
**Race**	Black	46,949 (6.3)	81,183 (6.8)	0.0307	49,290 (6.6)	78,452 (6.6)	0.0012
	Other	39,060 (5.2)	59,829 (5.0)		38,334 (5.1)	60,640 (5.1)	
	White	376,176 (50.3)	584,959 (49.1)		371,598 (49.6)	588,544 (49.6)	
	Unknown	286,158 (38.2)	464,353 (39.0)		290,336 (38.7)	459,958 (38.7)	
**Ethnicity**	Hispanic	37,233 (5.0)	62,511 (5.3)	0.0178	38,418 (5.1)	61,082 (5.1)	0.0006
	Non-Hispanic	624,209 (83.4)	985,057 (82.8)		622,353 (83.0)	985,808 (83.0)	
	Unknown	86,899 (11.6)	142,751 (12.0)		88,786 (11.8)	140,703 (11.8)	
**Insurance Type**	Commercial	317,681 (42.5)	522,369 (43.9)	0.0704	327,605 (43.7)	516,557 (43.5)	0.0051
	Medicaid	85,173 (11.4)	154,124 (12.9)		92,586 (12.4)	147,030 (12.4)	
	Medicare Advantage	279,592 (37.4)	410,047 (34.4)		263,539 (35.2)	419,911 (35.4)	
	Medicare FFS	14,509 (1.9)	21,622 (1.8)		13,940 (1.9)	22,074 (1.9)	
	Other	49,418 (6.6)	79,108 (6.6)		49,937 (6.7)	78,936 (6.6)	
	Unknown	1,970 (0.3)	3,054 (0.3)		1,950 (0.3)	3,085 (0.3)	
**Region**	Midwest	152,244 (20.3)	284,437 (23.9)	0.0891	170,570 (22.8)	269,236 (22.7)	0.0023
	Northeast	192,533 (25.7)	303,332 (25.5)		191,263 (25.5)	303,496 (25.6)	
	South	226,796 (30.3)	335,353 (28.2)		215,942 (28.8)	342,916 (28.9)	
	West	149,469 (20.0)	226,288 (19.0)		145,396 (19.4)	230,129 (19.4)	
	Unknown	27,301 (3.6)	40,914 (3.4)		26,386 (3.5)	41,815 (3.5)	

		Pre-weighting			Post-weighting		
		mRNA-1273.222	BNT162b2 Bivalent	SMD	mRNA-1273.222	BNT162b2 Bivalent	SMD
**Month of index**	08-2022	7 (0.0)	14 (0.0)	0.0679	9 (0.0)	13 (0.0)	0.0011
	09-2022	164,696 (22.0)	293,437 (24.7)		177,085 (23.6)	280,869 (23.7)	
	10-2022	261,030 (34.9)	414,468 (34.8)		261,220 (34.8)	413,664 (34.8)	
	11-2022	160,726 (21.5)	240,222 (20.2)		155,036 (20.7)	245,464 (20.7)	
	12-2022	101,945 (13.6)	151,691 (12.7)		98,156 (13.1)	155,425 (13.1)	
	1-2023	46,381 (6.2)	69,596 (5.8)		44,752 (6.0)	71,025 (6.0)	
	2-2023	13,558 (1.8)	20,896 (1.8)		13,298 (1.8)	21,133 (1.8)	
**Place of service**	IP claim	1,975 (0.3)	4,814 (0.4)	0.0758	2,626 (0.4)	4,196 (0.4)	0.0014
	OP EHR	5,138 (0.7)	7,510 (0.6)		5,021 (0.7)	7,843 (0.7)	
	OP claim	80,689 (10.8)	155,641 (13.1)		91,670 (12.2)	145,459 (12.2)	
	Pharmacy claim	660,541 (88.3)	1,022,359 (85.9)		650,240 (86.7)	1,030,096 (86.7)	
**Number of OP visits, mean (SD)**		1.8 (5.0)	1.7 (5.0)	0.0108	1.7 (5.0)	1.7 (5.0)	0.0008
**Number of hospitalizations, mean (SD)**		0.2 (0.8)	0.2 (0.9)	0.0159	0.2 (0.9)	0.2 (0.9)	0.0009
**Primary series COVID-19 vaccine**	Heterologous	63,925 (8.5)	132,120 (11.1)	0.1754	78,323 (10.4)	121,679 (10.2)	0.0066
	Homologous	223,982 (29.9)	269,224 (22.6)		187,906 (25.1)	298,539 (25.1)	
	Not reported	460,436 (61.5)	788,980 (66.3)		483,328 (64.5)	767,375 (64.6)	
**Time since last monovalent COVID-19 vaccine**	≤90 days	12,254 (1.6)	14,892 (1.3)	0.2316	10,377 (1.4)	16,532 (1.4)	0.0015
	91–180 days	157,894 (21.1)	160,558 (13.5)		121,634 (16.2)	192,265 (16.2)	
	>180 days	437,084 (58.4)	715,822 (60.1)		446,430 (59.6)	707,975 (59.6)	
	Not reported	141,111 (18.9)	299,052 (25.1)		171,115 (22.8)	270,821 (22.8)	
**Time since last COVID-19 infection**	≤120 days	32,182 (4.3)	52,073 (4.4)	0.0299	32,667 (4.4)	51,730 (4.4)	0.0007
	121–180 days	17,016 (2.3)	27,070 (2.3)		17,133 (2.3)	27,083 (2.3)	
	>180 days	66,145 (8.8)	115,378 (9.7)		70,366 (9.4)	111,495 (9.4)	
	Not reported	633,000 (84.6)	995,803 (83.7)		629,391 (84.0)	997,285 (84.0)	

		Pre-weighting			Post-weighting		
		mRNA-1273.222	BNT162b2 Bivalent	SMD	mRNA-1273.222	BNT162b2 Bivalent	SMD
**Patients with underlying medical conditions**	Asthma	106,345 (14.2)	173,045 (14.5)	0.0093	108,234 (14.4)	171,378 (14.4)	0.0003
	Cancer	98,649 (13.2)	149,769 (12.6)	0.0179	95,634 (12.8)	151,832 (12.8)	0.0008
	Cerebrovascular disease	67,396 (9.0)	103,404 (8.7)	0.0112	65,582 (8.7)	104,310 (8.8)	0.0012
	Chronic kidney disease	105,270 (14.1)	162,009 (13.6)	0.0132	102,392 (13.7)	163,070 (13.7)	0.0021
	Chronic lung disease[a]	97,671 (13.1)	148,729 (12.5)	0.0167	94,682 (12.6)	150,514 (12.7)	0.0013
	Chronic liver disease	14,144 (1.9)	22,665 (1.9)	0.0010	14,195 (1.9)	22,543 (1.9)	0.0003
	Cystic fibrosis	282 (0.0)	418 (0.0)	0.0013	269 (0.0)	427 (0.0)	<0.0001
	Diabetes type 1 or 2	248,850 (33.3)	383,323 (32.2)	0.0224	243,278 (32.5)	386,456 (32.5)	0.0018
	Disability	82,002 (11.0)	139,255 (11.7)	0.0234	86,182 (11.5)	136,041 (11.5)	0.0013
	Heart conditions	167,184 (22.3)	253,448 (21.3)	0.0254	161,512 (21.5)	256,842 (21.6)	0.0019
	HIV	7,206 (1.0)	11,201 (0.9)	0.0023	7,147 (1.0)	11,299 (1.0)	0.0002
	Mental health disorders	199,405 (26.6)	338,248 (28.4)	0.0396	208,868 (27.9)	330,272 (27.8)	0.0012
	Neurological conditions	26,484 (3.5)	46,607 (3.9)	0.0199	28,159 (3.8)	44,808 (3.8)	0.0009
	Obesity	252,077 (33.7)	405,503 (34.1)	0.0081	254,116 (33.9)	402,872 (33.9)	0.0004
	Primary immunodeficiencies	15,769 (2.1)	24,736 (2.1)	0.0020	15,521 (2.1)	24,716 (2.1)	0.0007
	Pregnancy[b]	4,998 (0.7)	9,447 (0.8)	0.0148	5,656 (0.8)	8,915 (0.8)	0.0005
	Physical inactivity	1,185 (0.2)	1,920 (0.2)	0.0007	1,208 (0.2)	1,909 (0.2)	0.0001
	Smoking[c]	151,537 (20.2)	241,868 (20.3)	0.0017	151,795 (20.3)	240,899 (20.3)	0.0008
	Solid organ or hematopoietic stem cell transplant	5,007 (0.7)	8,560 (0.7)	0.0060	5,214 (0.7)	8,312 (0.7)	0.0005
	Tuberculosis	435 (0.1)	710 (0.1)	0.0006	440 (0.1)	699 (0.1)	0.0001
	Use of immunosuppressants	63,093 (8.4)	97,056 (8.2)	0.0101	61,829 (8.2)	97,991 (8.3)	0.0001

IQR, interquartile range; SD, standard deviation; SMD, standardized mean difference

[a]except for asthma; [b]Includes recent pregnancy ; [c]Includes current and former smoker

Supplementary Methods

Calculating the standardized mean difference (SMD)

The SMD compares the difference in means in units of the pooled standard deviation. Unlike t-tests and other statistical tests of hypothesis, the SMD is not influenced by sample size. Thus, the use of the SMD can be used to compare the balance in measured variables between exposure groups. As such, SMDs were generated to assess differences in covariates before and after weighting. For continuous variables, the SMD is defined as:

$$d = \frac{(\bar{x}_{\text{treatment}} - \bar{x}_{\text{control}})}{\sqrt{\frac{s^2_{\text{treatment}} + s^2_{\text{control}}}{2}}}$$

Where $\underline{x}_{treatment}$ and $\underline{x}_{treatment}$ denote the sample mean of the covariate in the reference group (mRNA-1273.222) and the comparator exposure group (BNT162b2 Bivalent), respectively. While $\mathrm{s}^2_{\mathrm{treatment}}$ and $\mathrm{s}^2_{\mathrm{control}}$ denote the sample variance of the covariate in the reference group and the comparator exposure group, respectively. For dichotomous variables, the SMD is defined as:

Where $\hat{p}_{treatment}$ and $\hat{p}_{control}$ denote the prevalence or mean of the dichotomous variable in the

$$d = \frac{(\hat{p}_{\text{treatment}} - \hat{p}_{\text{control}})}{\sqrt{\frac{\hat{p}_{\text{treatment}}(1 - \hat{p}_{\text{treatment}}) + \hat{p}_{\text{control}}(1 - \hat{p}_{\text{control}})}{2}}}$$

reference group and the comparator exposure group. A threshold of 0.1 was used to determine covariate imbalance.
